# Kinetics of NH_3_ Desorption and Diffusion on Pt: Implications for the Ostwald Process

**DOI:** 10.1021/jacs.1c09269

**Published:** 2021-10-21

**Authors:** Dmitriy Borodin, Igor Rahinov, Oihana Galparsoro, Jan Fingerhut, Michael Schwarzer, Kai Golibrzuch, Georgios Skoulatakis, Daniel J. Auerbach, Alexander Kandratsenka, Dirk Schwarzer, Theofanis N. Kitsopoulos, Alec M. Wodtke

**Affiliations:** † Institute for Physical Chemistry, Georg-August University of Goettingen, Tammannstraße 6, 37077 Goettingen, Germany; ‡ Department of Dynamics at Surfaces, Max Planck Institute for Biophysical Chemistry, Am Fassberg 11, 37077 Goettingen, Germany; § Department of Natural Sciences, The Open University of Israel, 4353701 Raanana, Israel; ∥ Donostia International Physics Center (DIPC), Paseo Manuel de Lardizabal 4, 20018 Donostia-San Sebastián, Spain; ⊥ Kimika Fakultatea, Euskal Herriko Unibertsitatea UPV/EHU, P.K. 1072 Donostia-San Sebastián, Spain; # Department of Chemistry, University of Crete, 71003 Heraklion, Greece; ¶ Institute of Electronic Structure and Laser − FORTH, 71110 Heraklion, Greece; ▲ International Center for Advanced Studies of Energy Conversion, Georg-August University of Goettingen, Tammannstraße 6, 37077 Goettingen, Germany

## Abstract

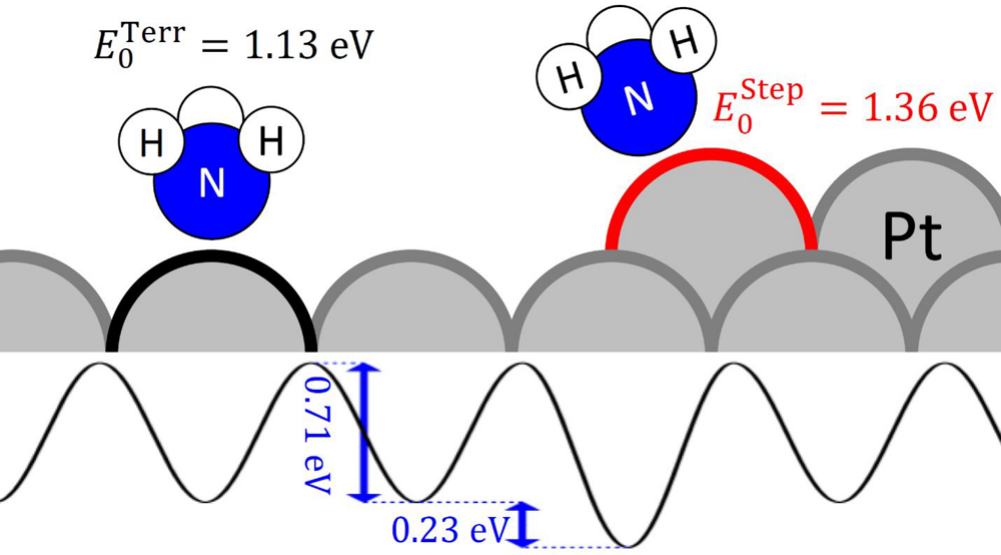

We report accurate time-resolved measurements of NH_3_ desorption from Pt(111) and Pt(332) and use these results to determine elementary rate constants for desorption from steps, from (111) terrace sites and for diffusion on (111) terraces. Modeling the extracted rate constants with transition state theory, we find that conventional models for partition functions, which rely on uncoupled degrees of freedom (DOFs), are not able to reproduce the experimental observations. The results can be reproduced using a more sophisticated partition function, which couples DOFs that are most sensitive to NH_3_ translation parallel to the surface; this approach yields accurate values for the NH_3_ binding energy to Pt(111) (1.13 ± 0.02 eV) and the diffusion barrier (0.71 ± 0.04 eV). In addition, we determine NH_3_’s binding energy preference for steps over terraces on Pt (0.23 ± 0.03 eV). The ratio of the diffusion barrier to desorption energy is ∼0.65, in violation of the so-called 12% rule. Using our derived diffusion/desorption rates, we explain why established rate models of the Ostwald process incorrectly predict low selectivity and yields of NO under typical reactor operating conditions. Our results suggest that mean-field kinetics models have limited applicability for modeling the Ostwald process.

## 1 Introduction

The Ostwald process is a critically important stepping stone for industrial production of artificial fertilizers, converting ammonia (NH_3_) to nitric acid (HNO_3_) in the presence of oxygen and water. The key to its success is the efficient oxidation of NH_3_ to nitric oxide (NO) on a Pt catalyst. In industry, the Ostwald process is conducted at temperatures of 1050–1250 K and total pressures between 1 and 12 bar with an ammonia to air ratio of 1:10.^1^ To initiate the oxidation, NH_3_ adsorbs with high probability to the majority terrace site and must then diffuse to low-coordination step sites, where it is able to react with oxygen.^2−8^ Thus, the competition between desorption and diffusion and the equilibrium between adsorption at step and terrace sites are critical factors in determining reaction probability; yet the competition between NH_3_ desorption and diffusion on Pt has never been investigated. There is not even an experimental consensus concerning such a basic parameter as the binding energy of NH_3_ at Pt(111). Molecular beam relaxation spectrometry (MBRS)^9^ yielded a binding energy of 0.68 eV, whereas analysis of collision-induced desorption (CID) experiments^10^ led to a value of 1.1 eV. Laser-induced desorption (LID)^11^ studies suggest a binding energy of ∼0.8 eV, consistent with results obtained with temperature-programmed desorption (TPD).^12,13^ Analysis of TPD data also reveals a weakening NH_3_–Pt bond with increasing NH_3_ coverage and contradicts the expectation one might infer from MBRS, conducted at low coverages, and CID, conducted at high coverages. Obviously, the uncertainty among the experimental determinations of the binding energy precludes any serious comparison with theory. This is presumably the reason why the NH_3_ binding energy at Pt(111), despite its importance, is still missing from experimental benchmark tables.^14^ Furthermore, real catalysts exhibit a diversity of active sites, including steps and kinks, and the relative binding strengths of molecules to different sites can determine the reactant’s ability to compete with other molecules to occupy the active site(s). Sadly, no reliable site-specific binding energies of NH_3_ on Pt have yet been reported.

The lack of reliable quantitative information concerning NH_3_/Pt interactions led to surrogate empirically optimized models, which unfortunately lack universality and transferability. Nevertheless, the Ostwald process has been modeled using transition state theory (TST) and density functional theory (DFT) to calculate the relevant rate parameters on single crystal model catalysts like Pt(111),^2,15^ Pt(100),^16^ and Pt(211)^2,4,17^ that are then used to elucidate the optimum process conditions. Such models have rarely been validated by comparison to experiment, of which there are few. One of the best known models, frequently used in reactor simulations of the Ostwald process, was developed by Kraehnert and Baerns^18^ (KB). The KB kinetics model relies on a mechanism derived from DFT calculations on Pt(111) by Offermans et al.^15^ and optimizing the rate parameters to achieve agreement with the experimental rates of NH_3_ oxidation on polycrystalline Pt at 1 mbar and 600 K. Experimental observations could only be explained assuming that adsorbed NH_3_ (NH_3_*) and O* occupy different binding sites. These sites were assigned to those found on Pt(111) single crystals—on top for NH_3_* and fcc hollow for O* and NO*.^18^ Other structural features like steps, which are known to be more reactive than terraces,^2,6,7^ were not considered. Scheuer et al. pointed out that the KB mechanism lacks quantitative transferability to the ammonia slip^19^ regime, where NH_3_ reacts with O_2_, forming predominantly N_2_ and H_2_O. The lack of transferability is likely due to the use of rate constants, which do not reflect the correct elementary processes. For example, the KB mechanism includes a 0.65 eV adsorption barrier for NO on Pt, in contradiction to our current understanding that NO–Pt adsorption is barrierless.^20,21^ Beyond this, the rate parameters used to describe NH_3_ desorption in the KB model include an unphysically low prefactor in the Arrhenius expression, suggesting the entropy of Pt-adsorbed NH_3_ is higher than that of the gas-phase molecule. Clearly, there is a pressing need for reliable information on site-specific binding energies and entropies of NH_3_ and other molecules on Pt surfaces.

In this work, we report elementary thermal rate constants for NH_3_ desorption from and diffusion on Pt(111) and Pt(332) at surface temperatures 430 ≤ *T* ≤ 620 K, derived from kinetic data obtained with the velocity-resolved kinetics method.^22^ We find that the kinetic traces for the desorption rate of NH_3_ from a Pt(111) surface do not follow first-order kinetics but are instead biexponential. This is attributed to the exceptionally high diffusion barrier of NH_3_ on the (111) terrace that slows down the diffusion across the terraces toward the steps: molecules that desorb from the terrace prior to reaching the steps comprise the fast component of the biexponential, whereas molecules that make contact with the steps comprise the slow component. We globally fit desorption data from Pt(111)—step density 0.4 ± 0.2% monolayer (ML)—and Pt(332)—step density 16.7%ML—using a kinetics model that includes NH_3_* desorption from terraces and steps, hopping across terraces and hopping from steps to terraces. From the derived rate constants, we can accurately compute the desorption rate of NH_3_ from Pt surfaces as well as the population of step and terrace sites as a function of step density, pressure, and temperature.

The high quality of the kinetic data over a wide range of temperatures provides a great deal of information through application of TST, but the most common implementations of TST reported so far cannot reproduce our results. This problem is solved by developing a semiempirical partition function of adsorbed ammonia that includes the coupling between several modes that actively participate in diffusion. Using this form of TST, we obtain an excellent fit to the measured rate constants as well as NH_3_’s binding energy on terraces of Pt(111) (1.13 ± 0.02 eV), the diffusion barrier between binding sites of Pt(111) (0.71 ± 0.04 eV), and the degree of energetic stabilization of NH_3_ at steps compared to terraces (0.23 ± 0.03 eV). These results are in good agreement with DFT calculations that we also report here. Note the diffusion barrier for this system is ∼65% of the binding energy, a strong violation of the so-called 12% rule,^23,24^ postulating that diffusion barrier constitutes a rather small fraction of a binding energy. Clearly, the 12% rule should be used with caution.

We also used DFT calculations to investigate the coverage dependence of the NH_3_ desorption rate; our results are able to reproduce previously reported TPD experiments carried out for NH_3_/Pt(111),^12^ conducted at much lower surface temperatures. This success of our approach over such a wide temperature range justifies modeling catalyst NH_3_ coverages at high temperatures and pressures typical for Ostwald catalysis reactors. Our model predicts NH_3_ coverages below ∼10%, whereas established reactor models predict fully covered catalysts at all conditions relevant to the Ostwald process. We believe this explains why some of the established reactor models tend to overestimate the degree of NH_3_ slippage at process-relevant conditions.^18^ Finally, we find that the derived desorption and diffusion rates from this work suggest that the mean-field approximation, frequently employed to model reaction rates, is not appropriate for description of NH_3_ reactivity on Pt under industrially relevant conditions.

## 2 Results

The velocity-resolved kinetics technique has been described in detail elsewhere.^22,25,26^ Compared to other kinetics methods applied to surface processes, it has the advantage of providing time-resolved desorption flux directly, as NH_3_’s velocity- and angle-resolved density is obtained as a function of its surface residence time using ion imaging. Briefly, NH_3_ is deposited at a Pt surface of known temperature using a short (∼35 μs) molecular beam pulse to initiate the thermal desorption process. The flux of desorbing ammonia (∝d[NH_3_^*^]/d*t*) is obtained as a function of residence time by scanning the delay between the molecular beam pulse and an ionization laser pulse. The beam-laser delay is easily converted to surface residence time through knowledge of the molecule’s speed. Together this yields the kinetic trace, defined as the flux of ammonia leaving the surface versus residence time. At each value of time, velocity-resolved kinetics provides not only the kinetic trace but also, in addition, the speed and angular distributions of the desorbing ammonia molecules. See section 5.1 for further details of the methods used for these measurements.

We obtained the speed distributions for NH_3_ desorption from both Pt(111) and (332) at several surface temperatures, *T*_*S*_ (Supporting Information (SI), section S1). We fit these to Maxwell–Boltzmann distributions, extracting an effective translational temperature for the desorbing molecules, *T*_tr_. For experiments with Pt(332), *T*_tr_ was found to be equal to *T*_*S*_; whereas, for Pt(111) *T*_tr_ was less than *T*_*S*_. Based on detailed balance,^27^ these results immediately indicate that NH_3_ adsorption to Pt has no activation barrier and, therefore, that the binding energy is equal to the desorption energy. Furthermore, we also obtain the shape of the sticking probability curve as a function of kinetic energy *S*(*E*_tr_), and by assuming *S*(*E*_tr_ = 0) to be 1,^28^ we obtain the absolute quantity *S*(*E*_tr_). This is used to obtain the thermal sticking probability ⟨*S*_0_⟩(*T*) between 0 and 2000 K, which is shown in section S2 in the SI. A previous report at a single temperature^9^ agrees well with our results.

**Figure 1:**
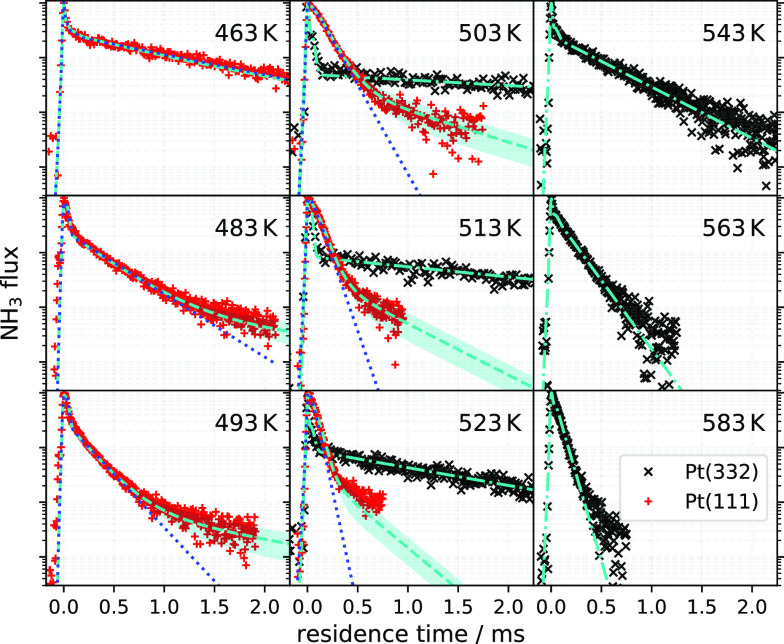
Kinetic traces of NH_3_ desorbing from Pt(111) (+) and Pt(332) (×) for surface temperatures between 463 and 583 K. The step density of the Pt(111) is 0.4 ± 0.2%ML and of Pt(332) is 16.7%ML. The light blue dashed (− −) and dash-dotted (− · −) lines show the global fit to the experimental kinetic data for Pt(111) and Pt(332), respectively. The shaded regions indicate the model uncertainty associated with the step density of the Pt(111) surface. The blue dotted line (···) indicates the model’s prediction for NH_3_ desorption rate from a step-free (ideal) Pt(111) surface.

We also obtained kinetic traces for NH_3_ desorption from Pt(111) and Pt(332), which are shown on a logarithmic scale at nine surface temperatures in Figure 1. The sharp, early time (first 0.1 ms) feature is a residual NH_3_ background from the directly scattered beam. It is independent of surface temperature and exhibits a narrow angular distribution peaking close to the specular angle. The dominant contribution to the observed signal is temperature-dependent and arises from thermally desorbing NH_3_. It has a broad angular distribution (∼cos(θ)), where θ is the angle with respect to the surface normal. The NH_3_ desorption rate from Pt(332) follows first-order kinetics as expected for a simple desorption process; however, NH_3_ desorbing from Pt(111) with 0.4 ± 0.2% steps is biexponential, with a fast (major) and a slow (minor) component. We repeated the desorption experiments at a Pt(111) surface with fewer steps and found that the slow component could be even further reduced (see section S3 in the SI). Notice that, when compared at the same temperature, the major component of Pt(111) data is faster than desorption from Pt(332). This indicates that NH_3_ has an increased residence time on highly stepped surfaces.

**Figure 2:**
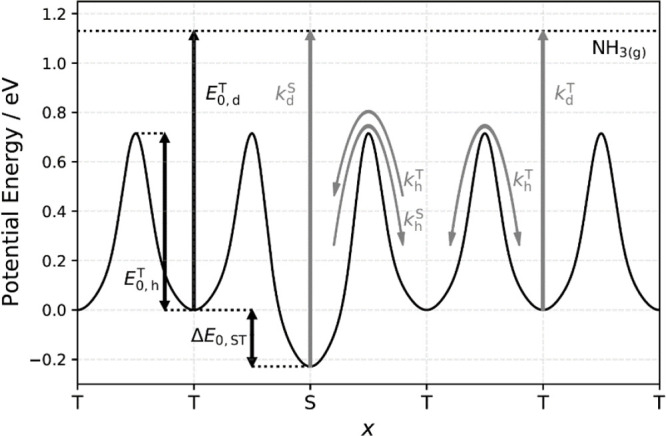
Schematic overview of the elementary processes (gray) included and energy parameters (black) extracted from the desorption–diffusion kinetics model. Steps and terraces are indicated by the letter S and T, respectively. The NH_3_ binding energy at (111) terraces of Pt is *E*_0,d_^T^ = 1.13 ± 0.02 eV, the site-to-site hopping barrier is *E*_0,h_^T^ = 0.71 ± 0.04 eV, and the energy preference for steps is Δ*E*_ST_ = 0.23 ± 0.03 eV. Following a similar strategy as described in ref (20), we include five elementary processes with first-order rate constants: (1) hopping between adjacent terrace sites, *k*_h_^T^; (2) hopping from terrace to step sites, which is assumed to be the same as *k*_h_^T^; (3) hopping from step to terrace sites, *k*_h_^S^; (4) desorption from terrace sites *k*_d_^T^; and (5) desorption from step sites, *k*_d_^S^. We note that *k*_d_^S^ describing process (5) is not an independent rate constant, *k*_d_^S^ = *k*_d_^T^*k*_h_^S^/*k*_h_^T^; see section S4.1 of the SI.

Figure 2 shows schematically the energy landscape and key elementary processes, with their rate constants, of a kinetics model capable of describing NH_3_ diffusion and desorption at Pt surfaces as a function of step density. The model is one-dimensional describing diffusion perpendicular to steps only. Each rate constant is parametrized in an Arrhenius form. The short time behavior of the kinetic trace, representing the direct scattering, is modeled with a temperature-independent model based on the arrival time distribution of the NH_3_ at the surface. We use periodic boundary conditions and make the model applicable to different step densities by varying the number of terrace sites separating the steps. Using this diffusion–desorption kinetics model (see section S4.2 of the SI for details), we fit the measured desorption rates from Pt(111) and Pt(332) simultaneously at all temperatures (see section S4.3 of the SI for details). The fit, shown as dashed (− −) and dash-dotted (− · −) lines in Figure 1, is excellent. The six independently derived Arrhenius rate parameters and their uncertainties are presented in Table 1.

**Table 1:** Rate Constants for Desorption and Diffusion of Ammonia on Platinum^a^,^b^

elementary rate constants	fitted parameters	fit results
*k*_d_^T^(*T*)	*E*_a,d_^T^/eV	1.09 ± 0.02
	log_10_(*A*_d_^T^/s^–1^)	14.8 ± 0.2
*k*_h_^S^(*T*)	Δ*E*_ST_/eV^c^	0.23 ± 0.03
	log_10_(*A*_h_^S^/s^–1^)	13.7 ± 0.6
*k*_h_^T^(*T*)	*E*_a,h_^T^/eV	0.73 ± 0.04
	log_10_(*A*_h_^T^/s^–1^)	13.6 ± 0.4
derived quantities		
*k*_d_^S^(*T*) = *k*_d_^T^*k*_h_^S^/*k*_h_^T^	*E*_a,d_^S^/eV	1.32 ± 0.04
	log_10_(*A*_d_^S^/s^–1^)	14.9 ± 0.6
*D*^T^(*T*)^d^	log_10_(*D*_0_^T^/cm^2^ s^–1^)	–1.9 ± 0.4

a Results were obtained from the global fit of the kinetics model to experimental desorption rates from Pt(111) and Pt(332).

b The elementary rate constants are parametrized according to the Arrhenius equation: *k*(*T*) = *A* exp(−*E*_a_/*k*_B_*T*).

c *E*_*a*,h_^S^ = *E*_*a*,h_^T^ + Δ*E*_ST_. Since *A*_h_^S^ ≈ *A*_h_^T^, the difference of activation energies Δ*E*_ST_ is nearly equal to the difference of binding energies Δ*E*_0, ST_.

d , where *D*_0_^T^ is derived from *A*_h_^T^ following ref (29).

Using the values of Table 1, we simulated how NH_3_ desorption would look in the absence of steps (blue dotted lines (···) in Figure 1). This shows that the fast component of the biexponential decay reflects direct desorption from terrace sites. In light of the relatively large step stabilization energy Δ*E*_ST_ also shown in Table 1, it becomes clear why desorption from Pt(332) is slower than the fast component of desorption from Pt(111). Table 1 also shows the computed prefactor for the terrace diffusion constant *D*^T^(*T*) derived from the hopping rate constant *k*_h_^T^(*T*) following ref (29) as well as the rate constant for direct desorption from steps *k*_d_^S^(*T*), which is derived from the other rate constants. Notice that the Arrhenius prefactor for terrace desorption and direct step desorption are nearly equal; that is, the entropy of the NH_3_ is nearly the same for these two binding sites. This is a striking result and means that the ammonia molecule is highly localized at terrace sites, a conclusion that is consistent with the large activation energy found for terrace hopping *E*_a,h_^T^ = 0.73 ± 0.04 eV.

Combining kinetic data with DFT parametrized TST can be highly useful. Thus, we performed a variety of DFT calculations using the Perdew–Burke–Ernzerhof (PBE) exchange-correlation functional.^30^ Our experiments are relevant to the zero-coverage limit; hence, we relied most heavily on DFT calculations carried out using a periodic 4 × 4 unit cell. We find that the on-top site is the most stable binding site for NH_3_* at Pt(111) with a (zero-point energy corrected) binding energy of 0.86 eV at 0.06 ML. In addition, we performed calculations with 2–4 NH_3_* molecules placed in the cell to produce coverages from 0.12 to 0.25 ML. We calculated the NH_3_* binding energy at Pt(111) in each case and find it to decreases linearly with increasing coverage with a slope of α = −1.61 eV/ML. Based on this finding, we determine a zero-coverage binding energy of 0.95 eV (see section S5 of the SI). Similarly, we calculate the binding energy at Pt(332) and, by comparison to Pt(111), find that the step stabilization is 0.30 eV.

In addition to the binding energy calculations, we performed analysis of the minimum energy pathway for hopping between on-top binding sites of Pt(111). We use the climbing image nudged elastic band (CI-NEB) method^31^ to locate the TS that we found at the bridge site. We obtain a zero-point energy corrected hopping barrier of 0.70 eV (0.52 eV) for the 4 × 4 (2 × 2) supercell. We also performed calculations of the harmonic frequencies at the on-top most stable binding site and for the transition states found at the bridge site (see Table 2).

**Table 2:** Results of DFT Calculations Performed for This Work: Harmonic Frequencies for NH_3_* at the Most Stable Binding Site (On-Top) and on the Transition State (TS) for Hopping (Bridge) Obtained from a 4 × 4 [ 2 × 2 ] Supercell Using the PBE Exchange-Correlation Functional^a^

mode		calculated harmonic frequencies/cm^–1^	
*v*_i_ (*v*_gas_)	description	on-top	TS for hopping (bridge)
*v*_1_ (3a)	asym. stretch	3483.1 [3484.4]	3546.7 [3550.5]
*v*_2_ (3b)	asym. stretch	3481.5 [3482.8]	3540.1 [3545.6]
*v*_3_ (1)	sym. stretch	3356.8 [3342.7]	3400.6 [3397.4]
*v*_4_ (4a)	asym. bending	1572.5 [1551.3]	1583.3 [1586.8]
*v*_5_ (4b)	asym. bending	1571.5 [1549.7]	1581.3 [1577.9]
*v*_umb_ (2)	umbrella mode	1142.0 [1055.2]	930.0 [856.2]
	free *C*_3_-axis rotation	- [ - ]	- [ - ]
*R*_*x*_	frustrated rotation	672.7 [636.3]	325.8 [131.3]
*R*_*y*_	frustrated rotation	672.4 [636.3]	269.9 [109.5]
*T*_*z*_	hindered translation	357.8 [338.3]	127.5 [ 45.9 ]
*T*_*x*_	hindered translation	122.8 [109.5]	190.9*i* [176.4*i*]
*T*_*y*_	hindered translation	119.9 [109.5]	68.2 [-]

a The imaginary frequency in *T*_*x*_ at the TS emerges from the degeneracy with the hopping coordinate. In this work we numbered the internal modes of adsorbed NH_3_ from high to low frequency. The conventional nomenclature from gas-phase vibrational spectroscopy is provided in parentheses for convenience.

## 3 Further Analysis and Discussion

### 3.1 Two Approaches to the Adsorbate Partition Function

In this section, we analyze the derived thermal rate constants in terms of transition state theory. This allows us to derive fundamental quantities such as the desorption energy and the diffusion barrier height. We elaborate detailed expressions for the adsorbate partition function in two ways. The partition function is normally considered a product of partition functions for the individual degrees of freedom (DOFs). We show here that this uncoupled TST approach fails to reproduce our experimental results. We then introduce a partition function which makes a better accounting of the state count when some of the DOFs are coupled (coupled TST). Coupled modes are identified through DFT calculations of NH_3_’s minimum energy pathway for hopping where we find that hindered translation and frustrated rotational modes are actively participating in the site-to-site exchange. This is reflected by an increase of surface–molecule distance and tilting of NH_3_’s symmetry axis along the minimum energy pathway. Also the associated vibrational frequencies decrease at the transition state for hopping by a factor of 2 to 3 (see Table 2), reflecting their importance for accurate description of the adsorbate entropy. This approach allows us to explain the temperature dependence of the rate constants precisely over the entire temperature range. This then provides the most accurate energy barriers for NH_3_ site-to-site hopping and desorption from Pt(111) presently available.

The general expression for the TST rate constant for desorption or diffusion is
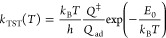
1where *Q*_ad_ is the partition functions of the ammonia adsorbate, *Q*^⧧^ is the partition function for the transition state, and *E*_0_ is the zero-point energy corrected barrier height. Highly accurate evaluation of *Q*_ad_ is often unnecessary in analyzing surface desorption rate data as the uncertainty in the experimental prefactor often exceeds an order of magnitude^32^ or in many cases is not measured at all.^8^ When analyzing high-quality kinetic data as obtained with velocity-resolved kinetics, *Q*_ad_ becomes a sensitive probe of the NH_3_/Pt interactions. Inappropriate approximations lead to detectable deviations from the measured desorption rates. In the following, we demonstrate the deficiencies of uncoupled TST and the advantages of coupled TST for the computation of *Q*_ad_.

The first approach, uncoupled TST (uTST), uses a sophisticated and established approach. Here, we base it on the approximation of a hindered translator,^33^ which is considered as one of the more accurate ways to compute *Q*_ad_.^34^ Here, the partition functions for *T*_*x*,*y*_ are described using a model potential, parametrized using DFT-calculated hindered translational frequencies (see Table 2) and the experimentally derived terrace hopping barriers (see section 3.3). The hindered translational partition function exploits an established interpolation scheme, ensuring its proper behavior at low and high temperatures.^21,33,35^ We treat NH_3_ rotation around its symmetry axis as a free rotation, justified by our DFT results and in agreement with previous theoretical work.^36^ The remaining DOFs are described by harmonic oscillators. For uTST, we use DFT-calculated frequencies for NH_3_ bound at its most stable binding site (see Table 2), consistent with how this approach is conventionally applied (for further details, see section S6.1 of the SI).

The uTST assumes that all DOFs are decoupled, making *Q*_ad_ a product of the partition functions of each DOF sensitive only to the structure of the molecule at the on-top binding site. However, when NH_3_* migrates over a diffusion barrier, its binding strength at the surface weakens, and consequently, the vibrational modes, especially those that strongly influence the oriented molecule–surface binding (*R*_*x*,*y*_, *T*_*z*_, and *v*_umb_), soften substantially (see Table 2). Since these modes have low frequencies, further frequency reduction has a large impact on the thermally accessible density of states.

To account for this effect, we developed a second approach, dubbed hereafter as the coupled TST (cTST) model. Briefly, cTST allows translation parallel to the surface to explicitly soften several of ammonia’s vibrational frequencies. This precludes a product form for *Q*_ad_. Instead, we construct a partition function where the vibrational frequencies of several modes (*v*_umb_, *R*_*x*_, *R*_*y*_, and, *T*_*z*_) vary along the minimum energy pathway for site-to-site hopping. This approach describes more faithfully the bond softening induced by the motion toward the diffusion barrier. Other DOFs are described as in uTST model. The construction of *Q*_ad_ for the cTST is described in detail in the SI section S6.2. In the next section, we apply uTST and cTST for the description of desorption and hopping rates of NH_3_ at Pt(111).

### 3.2 Analysis of the Desorption Rate Constants Using TST

To obtain desorption rate constants from TST, we must compute *Q*^⧧^. The modern formulation of TST prescribes a dividing plane that separates reactants from products such that every trajectory that originates in the reactant region of configuration space and evolves to the product region must pass through the dividing plane at least once. The choice of the position of the dividing plane can influence the probability for recrossing, which introduces a recrossing error to the TST rate. For NH_3_ desorption from Pt, it is convenient to place the dividing plane far from the surface, where the gas-phase NH_3_ molecule becomes the transition state. This choice of the transition state is convenient, as the thermal sticking coefficient ⟨*S*_0_⟩(*T*_S_) obtained above serves as the exact recrossing correction.^37^ Furthermore, *Q*^⧧^ is easily computed using tabulated gas-phase vibrational frequencies and rotational constants. We carried out this procedure in a similar way to a recent report for NO desorption from Pd;^21^ also, see section S6.3 of the SI.

**Figure 3:**
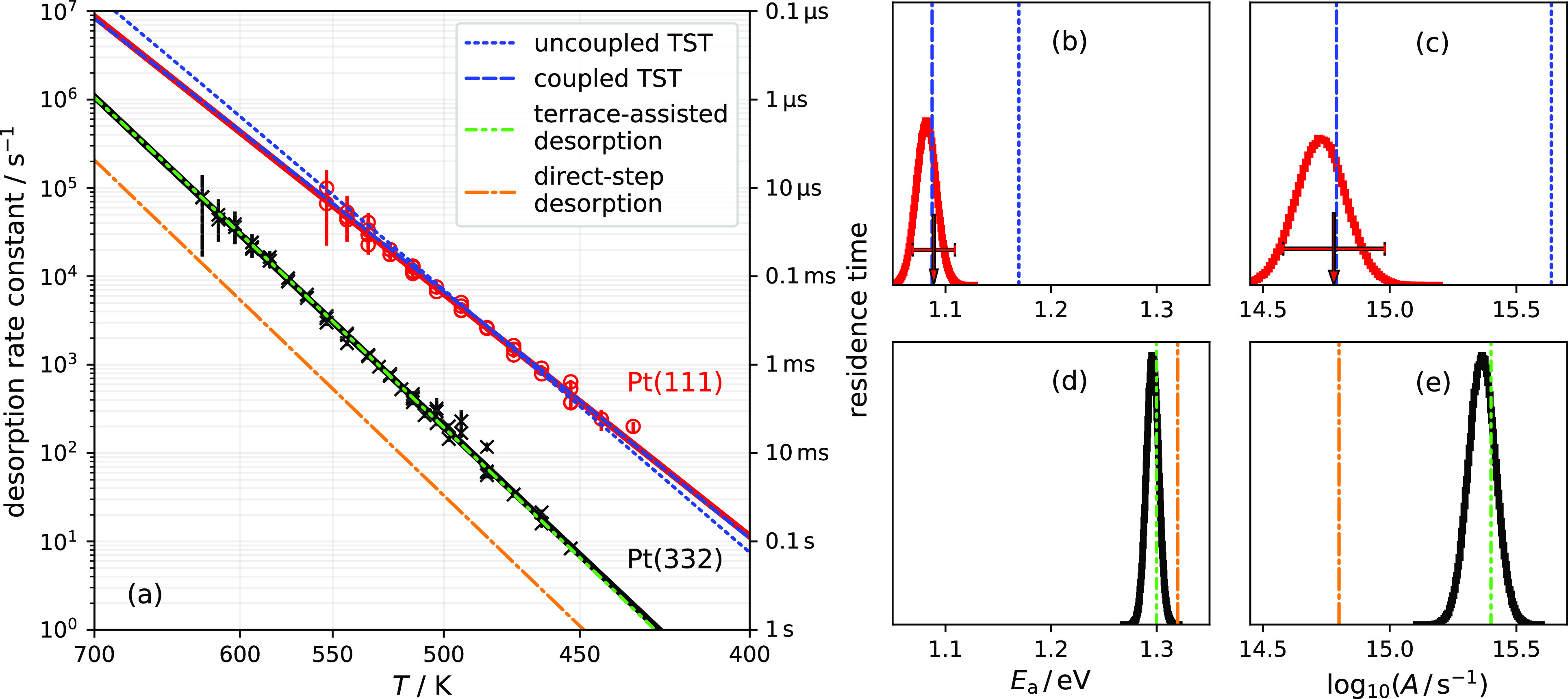
(a) NH_3_ desorption rate constants from Pt(111) terrace obtained from global kinetics model fit (red line) and from individual fits of the kinetic traces (red circles with error bars; see section S7 of the SI). The red line is *not* the Arrhenius fit to the circles. Terrace desorption rate constants are compared to the uTST (blue dotted) and cTST (blue dashed) models. The first-order desorption rate constants from Pt(332) (black crosses with error bars) and the corresponding Arrhenius fit (solid black line) are compared with a model assuming that desorption happens directly from steps (*k*_d_^S^ from Table 1, orange dash-dotted line) and a model that describes desorption as a “terrace-assisted” process including desorption from terraces and steps (eq 4, green dash-dot-dotted line). (b,c) Comparison of experimentally derived Arrhenius activation energy and prefactor for terrace desorption from Pt(111) and Arrhenius parameters predicted based on uTST (dotted blue line) and cTST (blue dashed line) models at 530 K (average temperature of present experiments). The red arrows with error bars result from global fit of diffusion–desorption kinetics model to experimental data (see SI section S4.3) and are represented by the red line in panel (a). The red histograms are parameter distributions emerging from Arrhenius fit (not shown for clarity) to red circles of panel (a). (d,e) Comparison of the Arrhenius parameter obtained from first-order desorption rate constants from Pt(332) to rate parameters based on direct-step and “terrace-assisted” desorption model at 530 K.

We may then write down a highly accurate formula for the experimentally derived desorption rate constants:
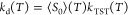
2Using eq 2, we optimized *E*_0_ to fit the cTST and uTST model to the experimentally derived terrace desorption rate constants—red circles with error bars and solid red line in Figure 3a. The red line is the terrace desorption rate constant that we extract from the global kinetics model fit (see also Table 1). Complementary to the global fit results, we analyze the fast decay of each NH_3_ kinetic trace from Pt(111) that we assigned to reflect the terraces’ desorption (see Figure 1) and derive the red circles from Figure 3a; see section S7 of the SI for details. The cTST (uTST) yields the blue dashed (dotted) line in Figure 3a with *E*_0,d_^T^ = 1.13 ± 0.02 (1.17) eV. Figure 3b,c offers a clear comparison of the cTST and uTST models to the derived Arrhenius rate parameters, which accurately represent the experimental rate constants. Here, the red arrow with the error bar represents the experimental uncertainty of the Arrhenius activation energy (Figure 3b) and Arrhenius prefactor (Figure 3c) for terrace desorption obtained from the global kinetics model fit to the experimental data, see section S4.3 of the SI for details. Complementing this, the red histograms display the uncertainty of the terrace desorption Arrhenius rate parameters that we derive from an Arrhenius fit (omitted in Figure 3a for clarity) to the red circles from Figure 3a. Both rate parameters are accurately reproduced by the cTST model (blue dashed vertical line), while the uTST model (blue dotted vertical line) clearly fails. Specifically, the uTST predicts an adsorbate entropy that is too low, and thus the resulting prefactor is too high (Figure 3c). The activation energy is then forced to be artificially high to compensate for this error in the prefactor (Figure 3b).

Based on this analysis, we recommend the results of the cTST model (*E*_0,d_^T^ = 1.13 ± 0.02 eV) for future use as the ammonia desorption energy on Pt(111). This value agrees with results from CID^10^ (1.1 ± 0.1 eV), although the error bar of that work was far outside chemical accuracy. Results from LID (0.8 eV) are clearly incompatible with the present work.^11^ This is likely due to the fact that those experiments were done at relatively high coverages. Despite working at low coverages, previous MBRS results (0.68 eV) are also incompatible with our results. The value of *E*_0,d_^T^ found with velocity-resolved kinetics is in poor agreement with DFT calculations when a PBE exchange-correlation functional is used—0.95 eV—see section S5 of the SI. Previous work with the PW91 functional^38^ yielded a value of ∼1.0 eV, which agrees only slightly better with the present results, confirming similarities between the PBE and the PW91 functionals.^14^

In the global fit of the kinetics model, we have also derived Δ*E*_ST_, the activation energy difference between desorption of NH_3_ at steps and terraces of Pt. As these two processes exhibit nearly the same prefactor, the difference of activation energies can be set equal to the difference in binding energies, Δ*E*_0,ST_ = 0.23 ± 0.03 eV. This compares well to previous TPD work (∼0.2 eV)^8^ and our DFT calculations, which predict an energy preference at steps of 0.3 eV. These results also have implications for the mechanism of desorption from steps. In Figure 3a, we compare a model that naively assumes desorption from a stepped surface, like Pt(332), that occurs directly—that is, diffusion from steps to terraces is unimportant. This clearly fails to capture the experimental observations (orange lines in Figure 3a,d,e). This suggests a more intricate step desorption mechanism, where both steps and terraces play a role. This is discussed further in section 3.5.1.

### 3.3 Analysis of the Hopping Rate Constants Using TST

**Figure 4:**
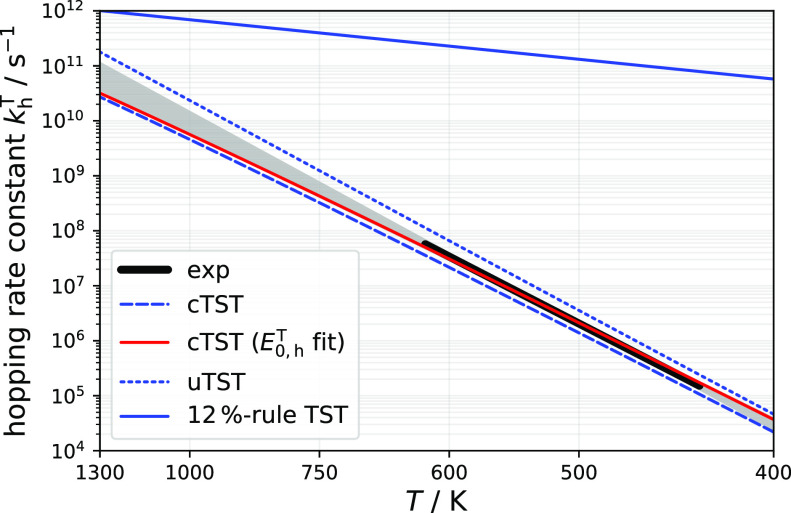
Broad black line shows the derived hopping rate constants in the temperature range of our experiments. The extrapolation of the derived hopping rate constant based on its Arrhenius parameters is shown as the gray shaded region that indicates the uncertainty of extrapolation. The blue solid line is the result of the hopping rate constant that is estimated based on the 12% rule (eq 3) suggested by Mavrikakis and co-workers.^23,24^ The blue dashed (dotted) line is the result of cTST (uTST) modeling of hopping rate constant using DFT-calculated hopping barriers. The red solid line is the cTST model using the hopping barrier fitted to the experimental rate constant. The residual mismatch between experiment and the cTST model can be explained with uncertainties in the assumptions of the TS partition functions (see text).

We also used the cTST and uTST models to describe NH_3_ site-to-site hopping on Pt(111). Here, we require the partition function of the hopping TS. To compute that, we approximate all but two DOFs as simple harmonic oscillators (with frequencies from Table 2). The exceptions are the NH_3_* rotation around its symmetry axis, which is again assumed to be a free rotation, and *T*_*y*_, which is treated as described in section S6.4 of the SI. Note that translation along *x* drops out, as this is the hopping coordinate. We first carry out this calculation using the DFT-derived and zero-point energy corrected hopping barrier of 0.70 eV presented above. The modeled cTST and uTST hopping rate constants at Pt(111) are shown as blue dashed and blue dotted lines of Figure 4, respectively, and are compared to the experimentally derived hopping rate constant (black solid line).

Again, cTST results are within ∼30% of the experimental values. The uTST model predicts rate constants that are systematically ∼2× too large. We note that the residual error in the cTST rate constant is not necessarily due to an error in the DFT barrier height. Instead, it could be an indication that a coupled partition function for the transition state is also required, something that is beyond the scope of this work. Coupling DOFs in the TS would increase TS state densities and increase the hopping rate constant, possibly leading to better agreement with the experiment. The use of an uncoupled partition function for the TS is also likely to be the reason why the deviation of uTST from experiment is only a factor of ∼2—due to a compensation of errors taking place in *Q*_ad_ and *Q*^⧧^.

We used two approaches to attempt an experimental determination of the hopping barrier. In the first, we optimized *E*_0,h_^T^ in the cTST model to fit our experimental hopping rate constant (black solid line of Figure 4). This led to 0.68 eV, which represents a lower limit. See also the red solid line in Figure 4. In the second approach, we used the DFT hopping barrier and determined the difference between activation energy and barrier height for hopping, based on the cTST model; *E*_a,h_^T^(500 K) – *E*_0,h_^T^ = 0.017 eV, which we subtracted from the experimentally obtained activation energy for hopping (*E*_a,h_^T^ = 0.73 ± 0.04; see Table 1). For the estimation of the activation energy, we used an average temperature of our Pt(111) experiments (500 K, which are most important to extraction of diffusion rates). This yielded an estimate of the hopping energy barrier: *E*_0,h_^T^ = 0.71 ± 0.04 eV, which also compares well with the DFT-derived hopping barrier obtained with the PBE functional and the estimated lower limit.

### 3.4 Comment about the 12% Rule for Diffusion Barriers

We notice that the Arrhenius expression-based prefactor derived for NH_3_ hopping—*A*_h_^T^ = 10^13.6±0.4^ s^–1^—is higher than values considered “common”, i.e., 10^<13^ s^–1^. However, this high value not only is in good agreement with DFT and cTST prediction of 10^13.3^ s^–1^ but also is physically reasonable. When a molecule is positioned at a weakly bound site, like the TS for hopping, its interaction with the surface is weakened, and thus the molecule is more likely to have an enhanced density of states and concomitant higher entropy. When the hopping barrier is very high, the TS is actually similar to a gas-phase molecule. Hence, the hopping prefactor will approach the prefactor for desorption, and the hopping of the molecule can be imagined to resemble transient or partial desorption, which is the case for NH_3_ on Pt(111). Contrasting this to the case of a small hopping barrier, the adsorbates’ density of states hardly changes at the hopping transition state compared to the molecule at its initial binding site. Therefore, the TST rate constant can be expressed using only the information about the molecule’s hindered translational frequency, here, ∼120 cm^–1^:
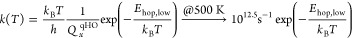
3The associated prefactor will be in the range of 10^12–13^ s^–1^, considered to be “typical”. A similar conclusion was reached by Mavrikakis and co-workers^23,24^ in developing the so-called 12% rule (=*E*_hop_/*E*_bind_) for diffusion barriers who argued that not only is the ratio of *E*_hop_ to *E*_bind_ likely to be about 0.12 but that the prefactors for hopping are commonly 10^12–13^ s^–1^. Ammonia binding to and diffusion on Pt(111) is an illustrative example, emphasizing that one has to be cautious with drawing universal conclusions about scaling relations based on stable site binding energies alone, without considering the nuances of molecular structure. In Figure 4, we show for comparison the hopping rate constant with barrier estimated based on the 12% rule with the corresponding hopping prefactor from eq 3, where the failure of this estimate becomes evident.

It had been realized earlier that the choice of the adsorbate entropy^33,34,39,40^ models and inclusion of anharmonic corrections^41^ has a substantial impact on the prediction of thermodynamic state functions relevant for the description of reaction rates. However, coupling of different DOFs, which we clearly show to be important for NH_3_ at Pt, is normally not considered. While these problems could, of course, be solved by demanding construction of a full dimensional potential energy surface, it is useful to develop a systematic hierarchy of correction schemes to provide an accurate description of thermal reaction rates using TST beyond the harmonic approximation. In that spirit, the application of the cTST model, incorporating the coupling of in-plane coordinates to different DOFs is a good step forward, especially as it requires little more input information from DFT than is already used for the less sophisticated approaches.

### 3.5 Implications for Modeling of the Ostwald Process

The ability of ammonia to find its way to steps is critical to it becoming chemically activated in the Ostwald process.^2,4,5,7^ In principle, this may happen by either direct adsorption and desorption to and from steps or by adsorption at terraces followed by diffusion to steps in competition with desorption. The complexity of the adsorption/diffusion/desorption often goes unappreciated. In this section, we take up this matter.

#### 3.5.1 Desorption Involving Multiple Active Sites

We have shown that NH_3_ desorption from Pt(111) is primarily due to desorption from terraces, whereas the desorption rate from Pt(332) is strongly influenced by steps. In Figure 3a, we show the extracted first-order rate constants of NH_3_ desorption from Pt(332) (black crosses) and compare them to the elementary rate constants for direct desorption from steps, *k*_d_^S^ (orange dash-dotted line)—direct desorption from steps fails to explain experiment. This is easily understood as molecules bound at steps can readily hop first to a terrace site, where desorption is much faster. Simply put, hopping from steps to terraces with subsequent desorption from terraces involves two low barrier processes, whereas direct desorption from steps involves one high barrier process. By assuming a steady-state concentration of ammonia at terraces (conditions that are ensured for Pt(332) experiments; see section 3.5.2) and including competitive desorption from terraces and steps, we can derive the effective first-order desorption rate constant of NH_3_ from a stepped surface (see section S8 of the SI for further details):
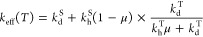
4where *k*_eff_(*T*) describes the “terrace-assisted” desorption of molecules (at low coverages) from surfaces with the step density, μ, which is defined as steps per unit cell length. The first term of eq 4 is the contribution of direct desorption from steps; the second term consists of a product between the hopping rate from steps to terraces, followed by the probability to desorb from terraces. For the derivation of this equation, we assumed that the total NH_3_ population at Pt(332) is well described by the population at steps. This assumption is justified, as NH_3_ has a high energy preference for steps, and entropic gain from binding at terraces is small due to a small number of terraces. See the SI section S8 for detailed derivation of this equation. The results of this “terrace-assisted” model are shown in Figure 3a as the green line, which agrees very well with the experimentally derived first-order desorption rate constants from Pt(332). In addition, the model reproduces the experimentally derived activation energy and prefactor for NH_3_ desorption from Pt(332) quite well (see Figure 3d,e).

Figure 3a shows that desorption rates from an Ostwald catalyst with multiple active sites cannot be adequately described if exchange between steps and terraces is not explicitly considered. However, very often, kinetics modeling of the desorption process does not consider multiple binding sites even though they are present at the stepped model catalysts. Most commonly, single binding sites are assumed that have the characteristic energies and prefactors that are associated with the most stable binding site.^2,17,18^ Clearly, this approach will underestimate the rate of actual desorption, where the adsorbate might exchange between binding sites and leave the surface through the less stable binding site. In fact, such errors, even if the rate constants are modeled correctly, may lead to erroneous conclusions about the efficiency of a catalyst at the desired reaction conditions.

#### 3.5.2 Limited Applicability of Mean-Field Approximation for NH_3_ Chemistry at Pt

We have observed that the NH_3_ desorption rate from Pt(111) with a step density of 0.4 ± 0.2% has a biexponential behavior, emerging from the competition between NH_3_ desorption from terraces and its slow diffusion to steps. At Pt(332), we observe a single-exponential desorption rate, indicating that NH_3_ equilibrates between steps and terraces, demonstrating that the competition between diffusion and desorption depends on the step density. Obviously, it will also depend on the temperature. Using the derived elementary process rate constants, we next investigate the competition between diffusion to steps and desorption from terraces as a function of the step density and temperature, including conditions relevant to Ostwald process.

**Figure 5:**
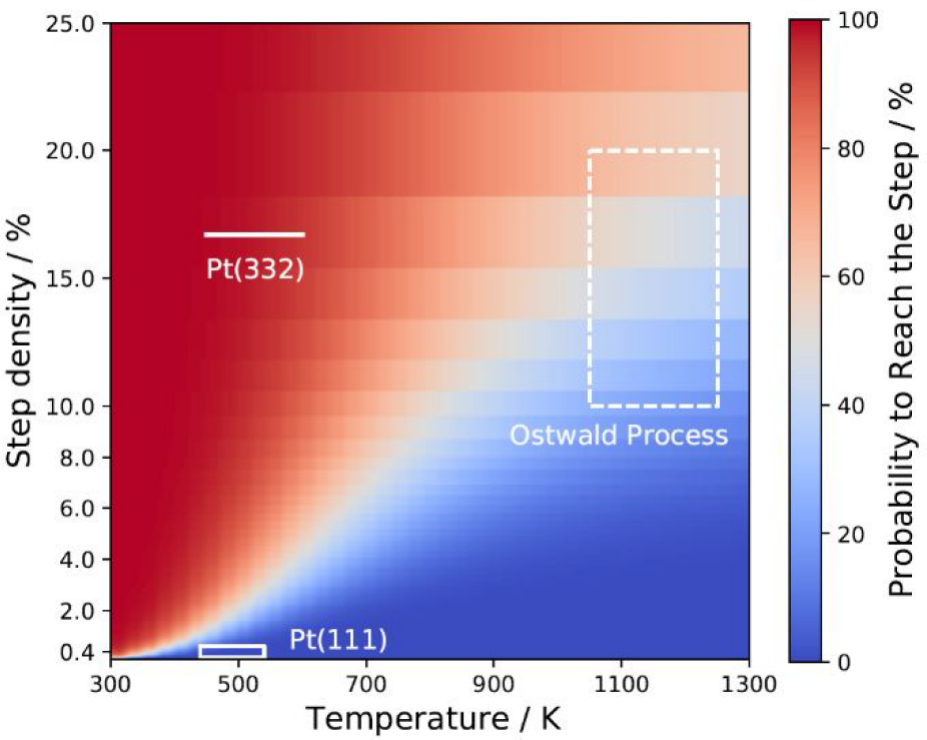
Probability of NH_3_ molecules that landed in the center of the terrace to reach the steps before desorption as a function of step density and catalyst temperature. The temperature ranges (1050–1250 K) and associated step densities of our experimental (solid box) and Ostwald process (dashed box) conditions are indicated in the plot. The step densities for the Ostwald catalyst are not known, but we consider typical step/edge densities found on catalytic nanoparticles^42,43^ as representative for real catalysts.

For the purpose of demonstration, we determine NH_3_’s probability to reach a step after landing at the center of a terrace. To do so, we place a low initial concentration of NH_3_ at the center of the terrace and solve the desorption–diffusion rate equations. We set *k*_h_^S^ = 0, “freezing” the NH_3_ molecule once it reaches a step. After all the NH_3_ molecules have either desorbed from the terrace or diffused to the steps, we determine the fraction of molecules that remained at steps. The results of this analysis are shown in Figure 5.

At low temperatures, diffusion to steps is fast compared to NH_3_ desorption, and all molecules can reach the step. At high temperatures, NH_3_ is less likely to reach the steps prior to desorption because the site-to-site hopping event approaches a time scale similar to that of the desorption event. Although the probability to reach the step increases with higher step densities, it can be clearly seen that at conditions typical for the Ostwald process not all molecules landing at majority terrace sites are able to reach the step prior to their desorption. It means that NH_3_ must adsorb on or very close to a step site in order to react. Under typical Ostwald conditions, the reactants (NH_3_* and O* at steps) are not able to encounter one another on a time scale faster than desorption, and as a consequence, the reactants cannot be assumed to be homogeneously mixed. This calls in question the basic assumption of the mean-field rate equations commonly used to model the Ostwald process.

The slow hopping rates and the previously observed preference for reaction at steps suggests that kinetics modeling of NH_3_ chemistry at Pt needs to explicitly account for different active sites and accurately describe the exchange between them—factors that have not been considered in kinetics modeling of Ostwald process so far. Notice that under catalytically relevant conditions, other adsorbates like NO* and O* will be present at the catalyst and likely decrease NH_3_’s mobility even further. These results suggest that the key reaction in the Ostwald process may, in fact, be diffusion-limited, contradicting current models that assume fast diffusion.^2,4,5,18^

#### 3.5.3 NH_3_ Coverages at a Pt Catalyst under Ostwald Process Conditions

Current kinetics models for NH_3_ oxidation at Pt lack transferability to reaction conditions different from those at which they were optimized. One possible reason for this is that the rate parameters employed do not describe elementary steps in the reaction. Using the experimentally derived desorption rates of this work, we can estimate the stationary NH_3_ isosteres as a function of temperature and pressure at steps and terraces of a stepped Pt catalyst at conditions typical for the Ostwald process. We compare those with predictions of the KB^18^ model that is frequently used for Ostwald process reactor simulations.^44−46^

This requires considering the coverage dependence of the ammonia desorption energy and prefactor. We use a coverage-dependent desorption barrier which we parametrize based on the experimentally derived NH_3_ binding energy (in the zero-coverage limit) and the scaling of the binding energy with coverage derived from DFT calculations (see sections S5 and S9 of the SI). We have performed harmonic frequency and hopping barrier calculations with DFT at 0.06 and 0.25 ML NH_3_ coverages, which allows us to estimate the coverage dependence of the prefactor. We assume that the logarithm of the prefactor—proportional to the entropy difference between the initial and transition state—scales linearly with coverage (see section S9 of the SI for further details). To test the constructed coverage-dependent desorption rate constant, we simulated TPD spectra from Pt(111) and compared them to results from previous works.^12^ Earlier TPD studies^12^ found broad NH_3_ desorption peaks, indicating substantial adsorbate–adsorbate interactions, influencing the desorption rate. We find that our model predicts the right temperature ranges for the TPD spectra and accounts correctly for the coverage dependence, which is reflected by the shape of the TPD trace (see section S9 of the SI).

**Figure 6:**
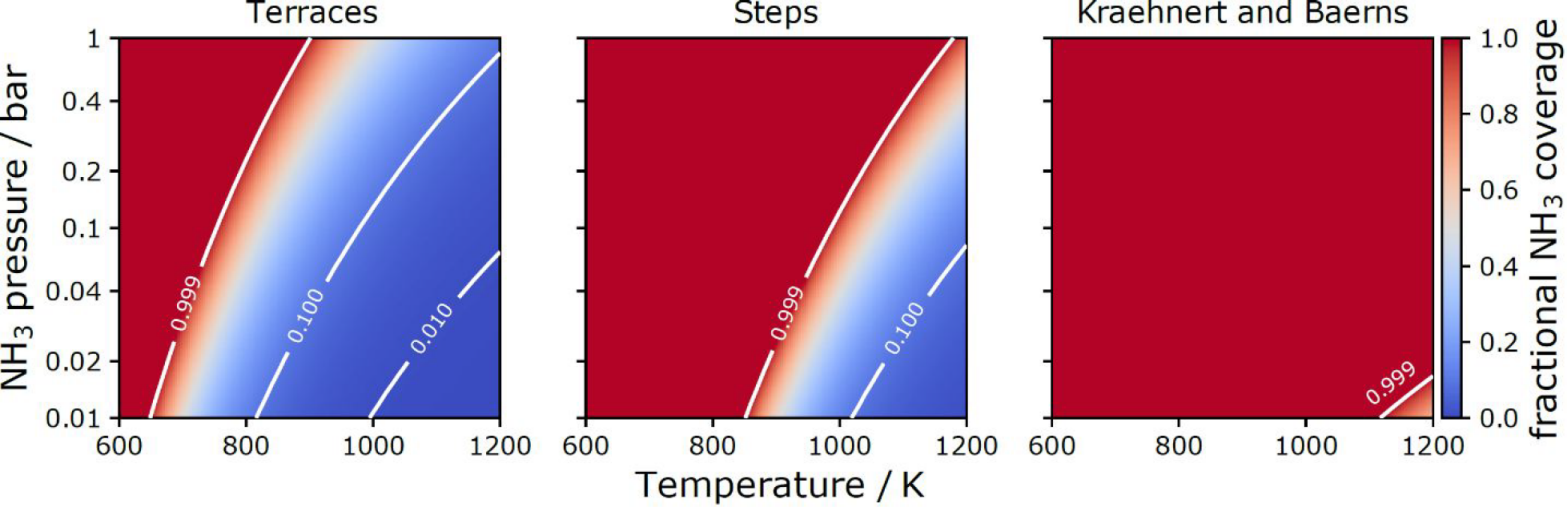
Fractional NH_3_ coverages at terraces (left) and steps (middle) of a Pt(332) model catalyst at temperatures and NH_3_ partial pressures typical for the Ostwald process. We compare our results (left and center panels) to the predictions of the KB model (right panel) which assumes one single active site for NH_3_. Note that the Ostwald process is conducted at total pressures of ≥1 bar with NH_3_ partial pressures of ∼10% of the total pressure.

Next, we used the desorption–diffusion model to determine the steady-state NH_3_ coverage at terraces and steps at surface temperatures and NH_3_ partial pressures characteristic of the Ostwald process (see section S9 of the SI for details). We chose the highly stepped Pt(332) surface as a model catalyst for the Ostwald process. The results are shown in Figure 6 and compared to the KB model predictions.

We find that the steady-state coverage of NH_3_ is strongly temperature- and pressure-dependent, whereas the KB model predicts saturated coverage under all conditions. Our model predicts rather low NH_3_ coverages (blue color in Figure 6) over a broad range of Ostwald process conditions. We hasten to point out that NH_3_ coverage will be strongly affected by coadsorbed O*, which can react to remove NH_3_ but also may induce stronger ammonia binding to the surface. Still the comparisons shown in Figure 6 suggest that it is likely that the commonly applied kinetics model overpredicts the coverage of NH_3_, which will lead to a higher degree of NH_3_ slippage (where less nitrogen ends up as NO—the desired product of the Ostwald process). This is consistent with the fact that the KB model under-predicts the NO yield and tends to overestimate the N_2_ yield at Ostwald process conditions.^18^

## 4 Summary and Conclusions

In this work, we have investigated the desorption kinetics of NH_3_ from Pt(111) and Pt(332) between 430 and 620 K using velocity-resolved kinetics. Detailed analysis of NH_3_ desorption kinetics using a diffusion–desorption kinetics model enabled us to extract rate constants for four elementary processes: direct desorption from terraces and from steps, site-to-site hopping at terraces, and hopping from steps to adjacent terrace sites. The measurement of a velocity-resolved kinetic trace provides simultaneously the speed distributions of desorbing molecules, from which we derive NH_3_ thermal sticking coefficients to Pt using the principle of detailed balance.

The rate constants of the elementary processes of desorption and diffusion have been further analyzed using TST with DFT input parameters. The conventional TST models, which describe the partition function of the adsorbate with uncoupled DOFs, fail to reproduce the experimental results. A correction scheme to the partition function is implemented that allows NH_3_ vibrational frequencies, associated with the molecule–surface interaction, to soften when displaced away from the most stable binding site. This approach faithfully reproduces the experimental kinetic data, and we derive accurate interaction energies for NH_3_ at Pt surfaces, which we summarize in Table 3.

**Table 3:** Most Important Results for Ammonia Interactions at Pt Surfaces

NH_3_/Pt interaction		recommended value
(111) desorption energy	*E*_0,d_^T^	1.13 ± 0.02 eV
(111) site-to-site hopping barrier	*E*_0,h_^T^	0.71 ± 0.04 eV
step preference over terrace	Δ*E*_0,ST_	0.23 ± 0.03 eV

Our work provides compelling evidence that NH_3_ diffusion on Pt(111) must pass over a large barrier, which is ∼65% of its binding energy. This is an exception to the so-called 12% rule. Instead of relying on such simple rules, our comparison with DFT calculations shows that the minimum energy path of diffusion appears to be highly accurate.

Having a quantitatively accurate kinetics model for ammonia desorption and diffusion, we were able to critically evaluate the approximations commonly employed in kinetics modeling of the Ostwald process. It is known from previous work that NH_3_ reacts efficiently with oxygen atoms at steps,^2,6,7^ while reaction at terraces is less efficient. We show that at temperatures typical for the Ostwald process, the NH_3_ hopping rate is close to its desorption rate, indicating that NH_3_ landing at terrace sites is unlikely to reach the steps, where it may react prior to its desorption. This implies that mean-field kinetics models have limited applicability for prediction of NH_3_ conversion rates and NO selectivity under Ostwald process conditions. Furthermore, by careful analysis of NH_3_’s desorption from Pt(332), we show that it is not possible to model the desorption rate from catalysts with multiple active sites by considering only the direct desorption from steps, an approach which is nevertheless persistently employed in kinetics modeling literature.^2,17^

With the help of DFT calculations, we extend the desorption rate constants beyond the zero-coverage limit of our experiment, which allows us to reproduce previously observed TPD spectra and to estimate NH_3_ coverages at Ostwald process conditions. The comparison of our results with a kinetics model commonly used for reactor simulations provides a simple explanation why established models tend to overpredict the extent of NH_3_ slip under Ostwald process conditions. We showed that this is a direct result of the model’s prediction of high NH_3_ coverages, which favor the formation of N_2_ and N_2_O and reduce the efficiency of NO formation.

In summary, the demonstrated approach exemplifies how the combination of high-quality kinetic data with TST analysis yields highly accurate elementary step rate constants, potentially capable of constructing mechanisms possessing high transferability without relying on empirical optimization within narrow range of experimental conditions.

## 5 Methods

### 5.1 Experimental

The Pt surfaces (MaTeck GmbH) were prepared by sputtering with Ar^+^ (3 keV) for 10 min and subsequent annealing at 1300 K for 20 min, and its cleanliness was verified with Auger electron spectroscopy. We employed two Pt(111) crystals with different step densities: the first had a step density quantified with atomic force microscopy of 0.4 ± 0.2% and the second a step density of 0.15 ± 0.05% estimated from the surface cut angle accuracy. We also used a Pt(332) crystal with a step density of 16.7%. Similar to previous work,^21,22,26^ a 20–50 μs long pulsed molecular beam of NH_3_ (0.5–2% NH_3_ in He) passed from the source chamber through two differential pumping chambers before entering a surface-scattering chamber, with a base pressure of 2 × 10^–10^ mbar. The incidence kinetic energy of NH_3_ in the beam was ∼0.25 eV. The NH_3_ pulses (repetition rate 20–40 Hz) strike the Pt at an incidence angle of 30° from the surface normal. The dose provided by each NH_3_ pulse was between 2 × 10^–4^ and 1 × 10^–3^ monolayer (ML).

Before investigating NH_3_ desorption from Pt, we verified that NH_3_ does not react under our conditions. We find no detectable H_2_ or N_2_ produced under our conditions. Isotopic exchange (e.g., NH_2_D and HD, using NH_3_ dosing of a D atom precovered Pt surfaces) was also absent. Furthermore, after dosing Pt(111) and Pt(332) surfaces with ∼2000 ML NH_3_, no nitrogen signal could be detected in the Auger spectrum.

The desorbing and the directly scattered NH_3_ were detected 2 cm from the surface using nonresonant multiphoton ionization (pulse duration 35 fs, average power 0.2 W, repetition rate 1 kHz). A pulsed homogeneous electric field, formed between two parallel flat meshes, projected the ions onto a time-gated MCP detector. The mass-to-charge ratio of the ions was selected with a time-gate on the microchannel plate (MCP), applied at a delay after pulsed extraction of the ions from the ionization region. The MCP amplified the ion signal, producing electrons that impinge upon a phosphor screen, emitting light recorded with a CCD camera. The pixel position provides information on the NH_3_ velocity, which is used to convert NH_3_ density to flux and to calculate the molecule’s flight times to the surface and from surface to the ionizing laser spot. We integrated the flux images from 400 and 1200 m/s at angles close to the surface normal, which strongly suppresses the background from direct scattering, which peaks at an angle of ∼30° and a velocity of 1500 m/s. This integral was determined at many beam laser delays, which we correct to surface residence time, *t*_res_, by subtracting the flight time, and we yield the kinetic trace d[NH_3_]/d*t* versus *t*_res_. The translational energy distribution of the molecules could be obtained by summing ion images over all measured timings.

A fraction of the NH_3_/Pt(332) data, from 453 to 553 K, was obtained in the 1 kHz detection setup and analyzed as has been described previously in detail.^25^

### 5.2 Computational

NH_3_ binding energies, diffusion barriers, and frequencies at Pt(111) and Pt(332) have been obtained using the Vienna Ab-initio Simulation Package.^47−51^ Periodic DFT calculations were performed at the level of generalized-gradient approximation using the PBE^30^ exchange-correlation functional.

The core–electron interactions were described by the projector-augmented wave potentials,^52,53^ with a cutoff energy of 400 eV for the plane-wave basis. The surfaces were modeled by a four-layer periodic slab, with each layer containing a 2 × 2 or a 4 × 4 supercell for Pt(111) and a 4 × 6 supercell for Pt(332). Two bottom layers were fixed during optimization. A 24 Å vacuum region was added to the slab to avoid interaction in the *z*-direction. The Brillouin zone was sampled with a 8 × 8 × 1 and a 5 × 5 × 1 with Γ-centered Monkhorst–Pack grids of special *k*-points for Pt(111) and Pt(332), respectively.

To predict adsorption energies, the two topmost surface layers and the NH_3_ molecule were allowed to relax until forces were lower than 0.02 eV/Å. Accounting for the same amount of DOFs, the reaction paths and transition states for diffusion and desorption were located by the CI-NEB method.^31^ The calculation was considered converged when forces were <0.05 eV/Å. The harmonic frequencies and normal modes were obtained solving the Hessian matrix for the DOFs of the NH_3_ molecule, applying two central finite differences with displacements of 0.02 Å.

## Supporting Information Available

NH_3_ speed distributions, analysis of sticking coefficients, comparison of desorption rates from Pt(111) with different step densities, equations for the diffusion–desorption kinetics model, coverage dependence of DFT-derived binding energies, construction of the partition functions for TST rate constant modeling, determination of first-order rate constants from experimental data, derivation of the terrace-assisted desorption model, construction of coverage-dependent desorption rate constants, and additional references (PDF)
