# Supplementary material for: Kinetics of NH3 Desorption and Diffusion on Pt: Implications for the Ostwald Process
Source: J Am Chem Soc. 2021 Oct 21;143(43):18305–16. doi: 10.1021/jacs.1c09269 (PMC8569812; doi:10.1021/jacs.1c09269)
Supplement: Supplementary file 1 — ja1c09269_si_001.pdf [file ja1c09269_si_001.pdf]

The Kinetics of NH3 Desorption and Diffusion on Pt: Implications for the Ostwald Process

Dmitriy Borodin[1,2], Igor Rahinov[3], Oihana Galparsoro[4,5], Jan Fingerhut[1], Michael Schwarzer[1], Kai Golibrzuch[2], Georgios Skoulatakis[2], Daniel J. Auerbach[2], Alexander Kandratsenka[2], Dirk Schwarzer[2], Theofanis N. Kitsopoulos[1,2,6,7]* and Alec M. Wodtke[1,2,8]**

[1]Institute for Physical Chemistry, Georg-August University of Goettingen, Tammannstraße 6, 37077 Goettingen, Germany.

[2]Department of Dynamics at Surfaces, Max Planck Institute for Biophysical Chemistry, Am Fassberg 11, 37077 Goettingen, Germany.

[3]Department of Natural Sciences, The Open University of Israel, 4353701 Raanana, Israel.

[4]Donostia International Physics Center (DIPC), Paseo Manuel de Lardizabal 4, 20018 Donostia-San Sebastián, Spain.

[5]Kimika Fakultatea, Euskal Herriko Unibertsitatea UPV/EHU, P.K. 1072 Donostia-San Sebastián, Spain.

[6]Department of Chemistry, University of Crete, Heraklion, Greece

[7]Institute of Electronic Structure and Laser – FORTH, Heraklion, Greece

[8]International Center for Advanced Studies of Energy Conversion, Georg-August University of Goettingen, Tammannstraße 6, 37077 Goettingen, Germany.

Email: *theo.kitsopoulos@mpibpc.mpg.de, **alec.wodtke@mpibpc.mpg.de

S1. Speed Distributions of desorbing NH3 from Pt(111) and Pt(332)

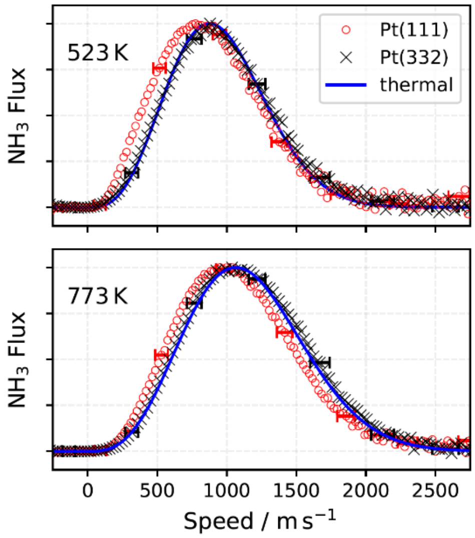

Figure S1: Speed distributions of desorbing NH_3 from Pt(111) (∘) and Pt(332) (×) detected at ±5° from surface normal at two surface temperatures. The horizontal error bar indicates the uncertainty in the speed calibration. The blue line is the thermal Maxwell-Boltzmann flux distribution at the temperature of the surface shown for comparison.

S2. Determination of the thermal sticking coefficient of NH3 at Pt(111)

The thermal sticking coefficient on Pt(111) is derived from the experimentally obtained translational energy distributions of desorbing NH_3 molecules using the principle of detailed balance. From detailed balance, the relation between the desorbing flux distribution (E, T_S) , the Maxwell-Boltzmann thermal (3D) distribution $F_{MB}(E, T_S)$, and the initial sticking probability function $S_0(E)$ is:

$$F(E, T_S) \propto S_0(E) F_{MB}(E, T_S), \tag{S1}$$

We observe that the desorbing NH_3 is subthermal, which indicates a decreasing sticking coefficient with increasing translational energy. The experimentally derived translational energy distributions of NH_3 desorbing from Pt(111) at 473-873 K are fitted globally to an empirical temperature independent $S_0(E)$. After fitting, the resulting sticking probability is obtained.

$$S_0(E) = 0.40 \times \exp(-30.95\ E/\text{eV}) + 0.60 \times \exp(-1.160\ E/\text{eV}). \tag{S2}$$

The corresponding fits are shown in Fig. S2.

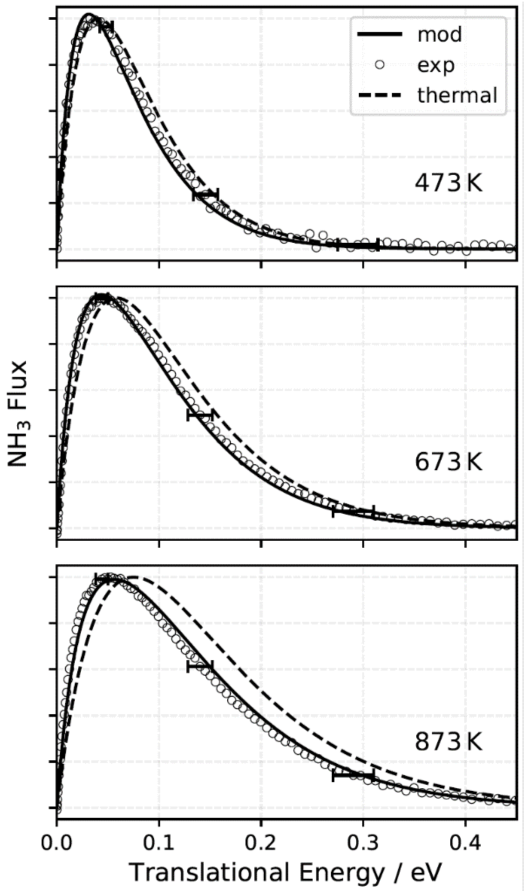

Figure S2: Experimentally derived translational energy distributions of NH_3 desorbing from Pt(111) are shown for surface temperatures between 473 and 873 K. The horizontal error bars indicate the uncertainty of the translational energy measurement. The black dashed line is the thermal Maxwell-Boltzmann distribution at the surface temperature. The black solid lines are the fit results of Eq. S1 and S2.

This approach gives the shape but not the absolute value of the sticking probability curve. We establish the absolute value using $S_0(E = 0) = 1$, an excellent assumption for a non-activated adsorption system like this one[1]. Figure S3 (left panel) shows the $S_0(E)$ found in this way. The thermal sticking coefficient $\langle S_0 \rangle(T)$ is then obtained from $S_0(E)$, by integrating over the Maxwell-Boltzmann energy distribution at temperature T:

$$\langle S_0 \rangle(T) = \frac{\int_0^\infty S_0(E) F_{MB}(E, T) \mathrm{d}E}{\int_0^\infty F_{MB}(E, T) \mathrm{d}E}. \tag{S3}$$

This is shown in Fig. S3 (right panel) along with the a previous report of $\langle S_0 \rangle(300\ \mathrm{K})$[2].

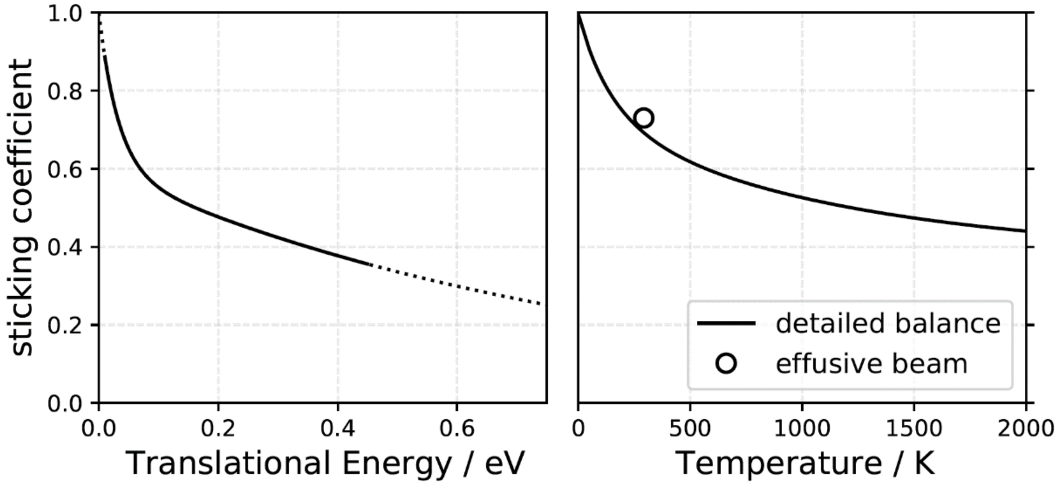

Figure S3: The sticking coefficient for NH_3 on Pt(111). Left panel as a function of NH_3's translational energy and right panel as a function of temperature. The empty circle is the experimental value of $\langle S_0 \rangle(T)$ found in Ref. [2]. The solid line in left figure shows the region over which the data constrains the fit. The NH_3 sticking coefficient on Pt(332) is unity for all translational energies and temperatures.

S3. Comparison of NH3 desorption rate from Pt(111) with different step densities

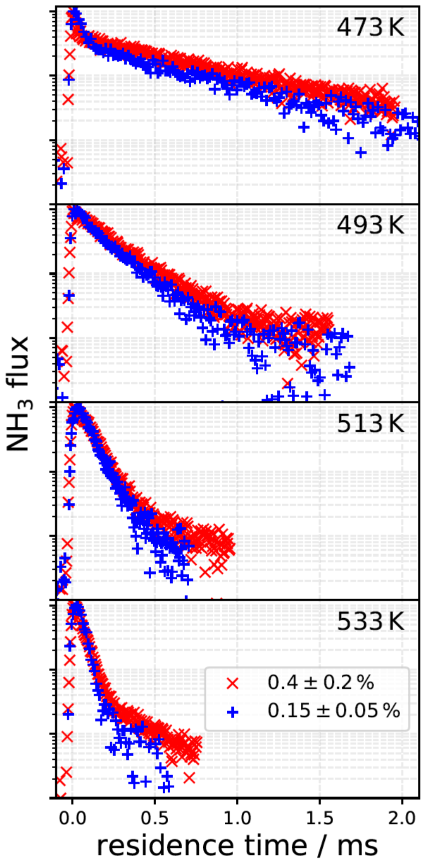

Figure S4: The kinetic traces of NH_3 desorption are shown for two Pt(111) crystals with different step densities: 0.15 ± 0.05 % (+) and 0.4 ± 0.2 % (×). At reduced step density the trace resembles more closely a single exponential. This shows that the slow component of the bi-exponential kinetic trace arises due to NH_3 interactions with steps.

S4. The Diffusion-Desorption Kinetic Model

S4.1. The relationship between k_d^S, k_h^T, k_h^S and k_d^T

The elementary rate constant for direct desorption from steps k_d^S can be obtained from the other three elementary rate constants, k_h^T, k_h^S, and k_d^T. Consider the TST rate expressions for these three elementary processes:

$$k_\mathrm{h}^\mathrm{T} = \frac{k_\mathrm{B}T}{h}\frac{Q_\mathrm{hop}^\ddagger}{Q_\mathrm{T}}\exp\left(-\frac{E_\mathrm{0,h}^\mathrm{T}}{k_\mathrm{B}T}\right), \tag{S4}$$

$$k_\mathrm{h}^\mathrm{S} = \frac{k_\mathrm{B}T}{h}\frac{Q_\mathrm{hop}^\ddagger}{Q_\mathrm{S}}\exp\left(-\frac{E_\mathrm{0,h}^\mathrm{T}+\Delta E_\mathrm{0,ST}}{k_\mathrm{B}T}\right), \tag{S5}$$

$$k_\mathrm{d}^\mathrm{T} = \frac{k_\mathrm{B}T}{h}\frac{Q_\mathrm{des}^\ddagger}{Q_\mathrm{T}}\exp\left(-\frac{E_\mathrm{0,d}^\mathrm{T}}{k_\mathrm{B}T}\right). \tag{S6}$$

Here, Q_T and Q_S are the partition functions for NH_3 adsorbed at terraces and steps, respectively, while $Q_\mathrm{hop}^\ddagger$ and $Q_\mathrm{des}^\ddagger$ are the TS partition functions for hopping and desorption, respectively. The following can then easily be shown

$$k_\mathrm{d}^\mathrm{T}\frac{k_\mathrm{h}^\mathrm{S}}{k_\mathrm{h}^\mathrm{T}} = \frac{k_\mathrm{B}T}{h}\frac{Q_\mathrm{des}^\ddagger}{Q_\mathrm{S}}\exp\left(-\frac{E_\mathrm{0,d}^\mathrm{T}+\Delta E_\mathrm{ST}}{k_\mathrm{B}T}\right) = k_\mathrm{d}^\mathrm{S}. \tag{S7}$$

We recognize that, within our approximations, expression S7 is nothing else but the TST expression for direct desorption from steps. We notice that this approximation is only valid for our setup of the kinetic model as we assume that the TS for terrace hopping is the same as for hopping between terrace and step sites.

S4.2. Kinetic rate equations in a non-uniform spatial grid

Our approach follows closely the work reported in Ref. [3], where it was shown how to account for surface diffusion by sequential hopping events between adjacent binding sites. The method employs periodic boundary conditions. For a step density of 0.4% this requires solving 250 coupled differential equations numerically, which is time consuming especially when employed within an optimization routine designed to obtain fits to the experimental data. However, in order to describe diffusion accurately, we do not need to treat each terrace site individually. The only unique binding sites that must be treated individually are the steps—the individual binding sites at the terraces can be coarse-grained into spatial regions that include multiple atomic rows, where the diffusion equation is solved. This dramatically reduces the number of coupled differential equations that need to be solved. We use spatial grids with atomic rows close to the steps, which increase in size when approaching the center of the terrace.

The diffusion-desorption rate equations are as follows:

$$\begin{aligned}\dot{c}_0 = {} & S_0^\mathrm{S}(1-c_0)F_\mathrm{B}(t) - k_\mathrm{h}^\mathrm{S}c_0(1-c_{m-1}) - k_\mathrm{h}^\mathrm{S}c_0(1-c_1) + k_\mathrm{h}^\mathrm{T}c_{m-1}(1-c_0)\\ & + k_\mathrm{h}^\mathrm{T}c_1(1-c_0) - k_\mathrm{d}^\mathrm{S}c_0,\end{aligned} \tag{S8}$$

$$\begin{aligned}\dot{c}_1 = {} & S_0^\mathrm{T}(1-c_1)F_\mathrm{B}(t) - k_\mathrm{h}^\mathrm{T}c_1(1-c_0) - k_\mathrm{h}^\mathrm{T}c_1(1-c_2) + k_\mathrm{h}^\mathrm{S}c_0(1-c_1)\\ & + k_\mathrm{h}^\mathrm{T}c_2(1-c_1) - k_\mathrm{d}^\mathrm{T}c_1,\end{aligned} \tag{S9}$$

$$\begin{aligned}\dot{c}_2 = {} & S_0^\mathrm{T}(1-c_2)F_\mathrm{B}(t) - k_\mathrm{h}^\mathrm{T}c_2(1-c_1) - k_\mathrm{h}^\mathrm{T}c_2(1-c_3) + k_\mathrm{h}^\mathrm{T}c_1(1-c_2)\\ & + k_\mathrm{h}^\mathrm{T}c_3(1-c_2) - k_\mathrm{d}^\mathrm{T}c_2,\end{aligned} \tag{S10}$$

...

$$\begin{aligned}\dot{c}_i = {} & S_0^\mathrm{T}(1-c_i)F_\mathrm{B}(t) + \frac{2D^\mathrm{T}}{d_i(d_{i+1}+d_i)}c_{i+1}(1-c_i) - \frac{2D^\mathrm{T}}{d_i(d_{i+1}+d_i)}c_i(1-c_{i+1})\\ & - \frac{2D^\mathrm{T}}{d_i(d_i+d_{i-1})}c_i(1-c_{i-1}) + \frac{2D^\mathrm{T}}{d_i(d_i+d_{i-1})}c_{i-1}(1-c_i) - k_\mathrm{d}^\mathrm{T}c_i,\end{aligned} \tag{S11}$$

...

$$\begin{aligned}\dot{c}_{m-1} = {} & S_0^\mathrm{T}(1-c_{m-1})F_\mathrm{B}(t) - k_\mathrm{h}^\mathrm{T}c_{m-1}(1-c_0) - k_\mathrm{h}^\mathrm{S}c_{m-1}(1-c_{m-2})\\ & + k_\mathrm{h}^\mathrm{T}c_{m-2}(1-c_{m-1}) + k_\mathrm{h}^\mathrm{S}c_0(1-c_{m-1}) - k_\mathrm{d}^\mathrm{T}c_{m-1}.\end{aligned} \tag{S12}$$

NH_3's fractional occupation of the binding site is given by c. Index i=0 indicates the step site, while indices 1 and $m-1$ indicates the terrace sites adjacent to the step. Indices 2 until $m-2$ are terrace sites. In the first term F_t is the flux of

molecules impinging on the surface, which is described by the molecular beam dosing function. For terrace sites, we use the sticking probabilities (S_0^{T}) from Fig. S3 and for step sites, we set the sticking probability (S_0^{S}) to unity. This is justified as the measured speed distributions of NH_3 desorbing from Pt(332) is thermal with $T_{\mathrm{tr}} = T_{\mathrm{S}}$ and exhibits a $\cos(\vartheta)$ angular distribution. In the equations above, $k_{\mathrm{h}}^{\mathrm{T/S}}$ are the hopping rate constants between adjacent atomic rows and D^T is the diffusion constant for NH_3 at terraces. The terms in parentheses are needed to account for effect of binding site occupation.

The terrace sites further away from steps are treated in non-uniform spatial elements with the width d_i. The definition of the spatial elements is shown in Fig. S5(a). In these regions, the terrace diffusion constant D^T is used. The resulting diffusion equations are in agreement with previously reported schemes[4]. We compare the desorption rates emerging from our non-uniform grid model to the model with only atomic rows, as suggested in Ref. [3], using a broad range of rate parameters and choose appropriate grids to reach a relative accuracy of at least 0.04% when modelling the desorption rate. This allows especially for low step density crystals, to reduce the evaluation time to a minimum, while having accurate results suitable for the kinetic model to fit the experimental data.

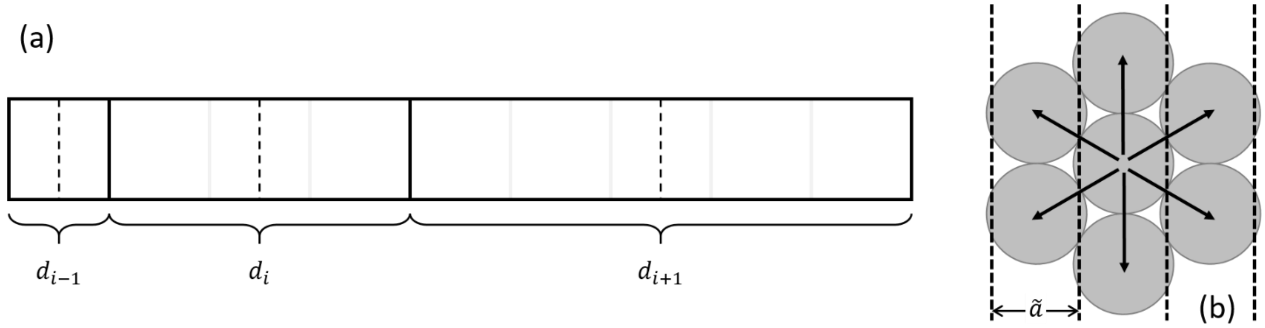

Figure S5: (a) Width definitions used for construction of diffusion equations in a non-uniform grid. (b) Sketch of degeneracy of pathways on a (111)-facet with black dashed lines indicating the step alignment on a Pt(332) crystal.

The diffusion constant D^T can be derived from the hopping rate constant between adjacent atomic rows following Ref. [5] or from the well-known formula for mean-square displacement. However, we have to account for the fact that hopping between adjacent rows on a Pt(111) will have degenerate pathways, as sketched in Fig. S5(b). Thus, D^T is given by:

$$D^T = \tilde{a}^2 k_h^T, \tag{S13}$$

where $\tilde{a}$ is the distance between two adjacent atomic rows (see Figure S5). When the width of spatial elements, d_i, is expressed in units of atomic rows as:

$$d_i = \tilde{a} n_i. \tag{S14}$$

Then Eq. S11 is reduced to:

$$\begin{aligned}\dot{c}_i = S_0^{\mathrm{T}}(1 - c_i)F_t + \frac{2k_h^T}{n_i(n_{i+1} + n_i)} c_{i+1}(1 - c_i) - \frac{2k_h^T}{n_i(n_{i+1} + n_i)} c_i(1 - c_{i+1}) \\ - \frac{2k_h^T}{n_i(n_i + n_{i-1})} c_i(1 - c_{i-1}) + \frac{2k_h^T}{n_i(n_i + n_{i-1})} c_{i-1}(1 - c_i) - k_d^T c_i.\end{aligned} \tag{S15}$$

The differential equations are solved following the protocol described in Ref. [6] and the corresponding desorption flux is obtained from:

$$F_t^{\mathrm{NH_3}} = k_d^S c_{0,t} + \sum_{i=1}^{m-1} n_i k_d^T c_{i,t}\,. \tag{S16}$$

S4.3. Fitting procedure and uncertainty estimation

The global optimization routine is based on least-square minimization of the residual, error-weighted difference between experiment and model – see Ref. [1] for details. After the initial guess of the six globally optimized rate parameters the global fitting employs a cyclical two step procedure.

1. First, the desorption rate is numerically calculated (Eq. S16) based on the initial guess or the optimized rate parameter values established in the previous execution of step 2. Then, the amplitudes of the direct scattering and the trapping-desorption channel are fitted (locally) to each individual kinetic trace. For description of direct scattering we use a function proportional to the incident molecular beam $F_{\mathrm{B}}(t)$ in Eq. S8 and Eq. S9. The residual over the total dataset is calculated.
2. The local fitting procedure of the direct-scattering and trapping desorption amplitudes ensures the lowest possible residual or the present set of rate parameters. The rate parameters are modified following the least squares procedure and step 1 is repeated until convergence of the residual is reached.

The uncertainties of the fitted parameters were estimated by displacing the rate parameter of interest from its best fit value in small increments, fixing it and re-optimizing the remaining five parameter globally until a noticeable mismatch (deviations beyond the noise level of the data) between the re-fitted model and the data was observed. The parameter values between which an accurate fit could be obtained define the uncertainty range (see arrow with error-bar of Figure 3(b) and 3(c)). From the present analysis, we find that the strongest parameter correlation is the correlation between A and E_{a} of the individual rate constants (increasing prefactor leads to increasing activation energy). We also find a strong correlation between $A_{\mathrm{h}}^{\mathrm{T}}$ and $A_{\mathrm{h}}^{\mathrm{S}}$ (which have an almost constant ratio). Correlations for other parameters are less pronounced. For terrace desorption (see Figure 3(b) and (c)) the error analysis compares well with a complementary error estimation (see Section 3.2 of the main text) reflecting its reasonability.

S5. Coverage dependence of NH3 binding energies at Pt(111)

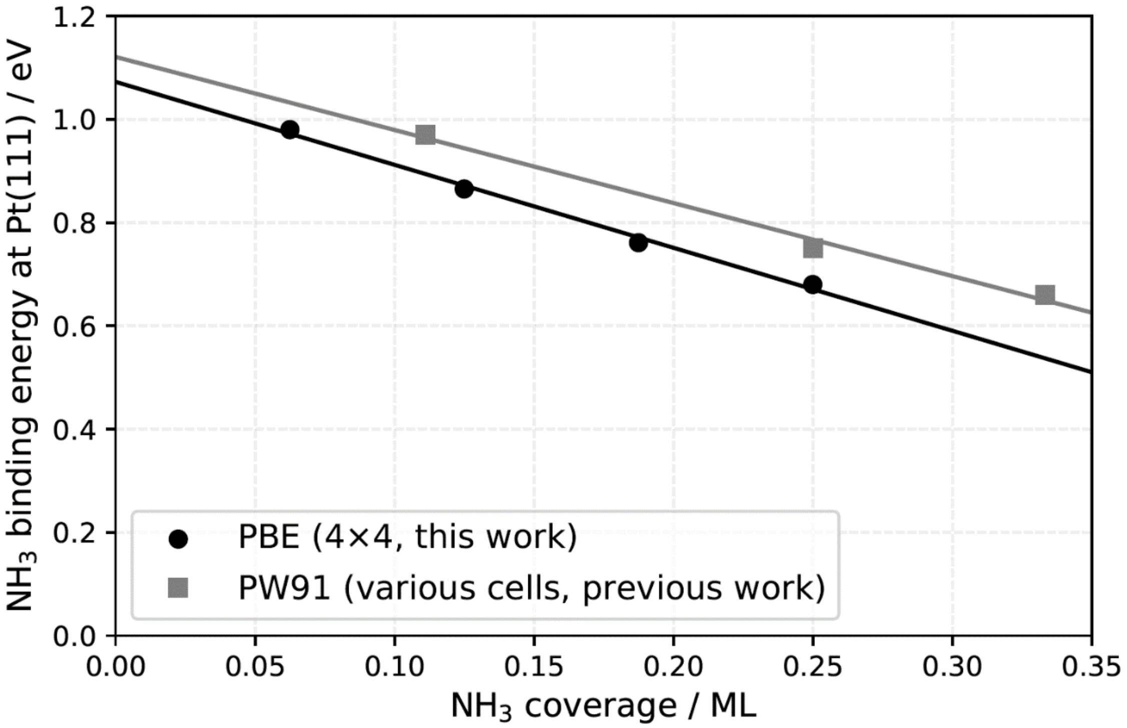

Figure S6: Calculated coverage dependence of NH_3 binding energies at Pt(111). The black points were obtained by placing 1, 2, 3 and 4 molecules inside a 4×4 unit cell and minimizing the energy. DFT energies were calculated with the PBE functional (the zero-point energy amounts ~0.12 eV at 0.06 ML but is not included for the energies shown in the plot). The grey squares are results from previously reported DFT calculations with the PW91 functional[7]. While the energies are different, the slopes of the fitted black and grey lines are very similar. The lines are linear extrapolations to zero-coverage for comparison to results derived from velocity-resolved kinetic experiments. The zero point energy correction on the binding energy at 0 and 0.06 ML, given in the main text, is included based of calculated frequencies at 0.06 ML. The scaling of the binding energy with the coverage was used for TPD and for isotherm simulations.

S6. The partition functions used in this work for TST modelling

S6.1. NH3* - uncoupled partition function for uTST model

The uncoupled TST (uTST) model used in this work employs the following adsorbate partition function $Q_{\mathrm{ad}}^{\mathrm{uTST}}$

$$Q_{\mathrm{ad}}^{\mathrm{uTST}} = \left(\prod_{i=1}^{5} Q_{v_i}^{\mathrm{qHO}}\right) Q_{v_{\mathrm{umb}}}^{\mathrm{qHO}} Q_{R_x}^{\mathrm{qHO}} Q_{R_y}^{\mathrm{qHO}} Q_{T_z}^{\mathrm{qHO}} Q_{C_3}^{\mathrm{rot}} \times \\ \frac{2\pi m k_{\mathrm{B}} T}{h^2} \int_{-a/2}^{a/2} \int_{-b/2}^{b/2} \exp\left(-\frac{U_{n,\gamma}(x,y)}{k_{\mathrm{B}}T}\right) \mathrm{d}x\mathrm{d}y \left(\frac{Q_{T_x}^{\mathrm{qHO}}}{Q_{T_x}^{\mathrm{cHO}}}\right)\left(\frac{Q_{T_y}^{\mathrm{qHO}}}{Q_{T_y}^{\mathrm{cHO}}}\right). \tag{S17}$$

Here the vibrational partition function of individual DOFs appear in an overall product form. NH_3*'s possesses six internal modes—v_{1-5} and v_{umb} that correlate to the normal modes of the gas-phase molecule. Due to adsorption to the surface, v_{umb} no longer inverts and two of rotational DOFs with rotation axes perpendicular to NH_3*'s symmetry axis, become frustrated rotations—R_x and R_y—and translation perpendicular to the surface—T_z—correlates to the external stretch of the adsorbate. All of these modes are treated as simple quantum harmonic oscillators,

$$Q^{\mathrm{qHO}} = \frac{1}{1-\exp\left(-\frac{h\nu}{k_{\mathrm{B}}T}\right)}, \tag{S18}$$

where, ν is the harmonic frequency of the mode at the most stable binding site (see Table 2 of the main text).

The rotation around NH_3* symmetry axis remains free, validated from previous work[8], and is described by the 1D-rotational partition function:

$$Q_{C_3}^{\mathrm{rot}} = \frac{\sqrt{\pi}}{\sigma}\sqrt{\frac{k_B T}{C}}, \tag{S19}$$

where C is the rotational constant and σ is the corresponding symmetry number.

NH_3*'s two translational DOFs parallel to the surface $T_{x,y}$ are treated within the hindered translator model[9-11], using a periodic interaction potential $U_{n,\gamma}(x,y)$, which we write in the following form:

$$U_{n,\gamma}(x,y) = f(x;n,\gamma) + f(y;n,\gamma). \tag{S20}$$

$f(x;n,\gamma)$ and $f(y;n,\gamma)$ contain adjustable parameters, n and γ, that are used to reproduce the harmonic frequency of T_x and T_y obtained from DFT, and the experimentally derived energy barrier for site-to-site hopping. To match both of these quantities, the commonly employed 1D model potential functions[9-10, 12] are better replaced by functions of the following form:

$$f(\xi;n,\gamma) = (1-\gamma)E_{0,\mathrm{h}}^{\mathrm{T}}\left(1-\cos^2\frac{\xi\pi}{a}\right) + \gamma E_{0,\mathrm{h}}^{\mathrm{T}}\left(1-\cos^2\frac{\xi\pi}{a}\right)^n, \tag{S21}$$

where, ξ is x or y, a is the length of the unit cell (2.77 Å), $E_{0,\mathrm{h}}^{\mathrm{T}}$ is the experimentally derived hopping barrier (0.71 eV). The hindered translation potential used in uTST $f(\xi; n = 2, \gamma = 0.47)$ is shown in Figure S7. The configuration integral above is classical and converges to 0 at low temperatures. We ensure proper quantum behavior of the partition function—i.e. $Q \geq 1$—at low temperature by including the term $Q_{T_\xi}^{\mathrm{qHO}}/Q_{T_\xi}^{\mathrm{cHO}}$. This correction term is described in Ref. [10] in detail.

S6.2. NH3* - coupled Q_{ad} for use in the coupled TST model

Despite energy sampling of a multidimensional potential energy surface for NH_3 at Pt(111) would be the most straightforward approach it is computationally demanding. Thus, we require clever approximative methods for the description of the potential energy going beyond the harmonic approximation and the assumption of normal modes.

Because the coupling of DOFs for partition functions in TST is not common and there are many ways this might be implemented, we first illustrate our idea using a 2D toy system. Consider an atom on a one-dimensional catalyst surface, which exhibits displacement over a diffusion barrier along x and harmonic vibration normal to the surface along z. For motion along x, the harmonic frequency in the z direction varies due to the changing interaction with the catalyst. This can be expressed as an x-dependent force constant $k_z(x)$, where the total energy expression is then approximated by:

$$H_{\mathrm{toy}}(p_x,p_z,x,z) = \frac{p_x^2}{2m} + \frac{p_z^2}{2m} + V(x) + \frac{1}{2}k_z(x)z^2. \tag{S22}$$

Here, the first two terms describe the kinetic energy along x and z, the third term describes the potential energy along the minimum energy pathway for diffusion, while the fourth term describes the potential energy for z displacement. This

formulation explicitly introduces coupling of x and z as k_z depends on x. The classical partition function for the toy system then becomes:

$$Q_{\mathrm{ad}}^{\mathrm{toy}} = \frac{1}{h^2} \int_{z=-\infty}^{\infty} \int_{x=0}^{a} \int_{p_z=-\infty}^{\infty} \int_{p_x=-\infty}^{\infty} \exp\left(-\frac{H_{\mathrm{toy}}(p_x, p_z, x, z)}{k_{\mathrm{B}}T}\right) \mathrm{d}p_x \mathrm{d}p_z \mathrm{d}x \mathrm{d}z, \tag{S23}$$

where a is the length of the unit cell. Combining Eq. S22 and S23 leads to:

$$Q_{\mathrm{ad}}^{\mathrm{toy}} = \frac{\sqrt{2\pi m k_{\mathrm{B}}T}}{h} \int_{-a/2}^{a/2} \exp\left(-\frac{V(x)}{k_{\mathrm{B}}T}\right) \frac{k_B T}{h v_z(x)} \mathrm{d}x = \frac{\sqrt{2\pi m k_{\mathrm{B}}T}}{h} \int_{-a/2}^{a/2} \exp\left(-\frac{V(x)}{k_{\mathrm{B}}T}\right) Q_z^{\mathrm{cHO}}(x) \mathrm{d}x. \tag{S24}$$

Here, $v_z(x)$ is the harmonic stretch frequency and the coupling between x and z manifests itself by a modification of the configurational integral over x, which now includes the z-coordinate stretch frequency in the x-dependent vibrational partition function $Q_z^{\mathrm{cHO}}(x)$. Since $k_B T \ll hv$, we replace $Q_z^{\mathrm{cHO}}(x)$ by its quantum mechanical counterpart:

$$\begin{gathered} Q_z^{\mathrm{cHO}}(x) = \frac{k_B T}{h v_z(x)} \\ \Downarrow \\ Q_z^{\mathrm{qHO}}(x) = \frac{1}{1 - \exp\left(-\frac{h v_z(x)}{k_B T}\right)}. \end{gathered} \tag{S25}$$

While this approach is certainly *ad hoc*, it represents a useful step beyond uTST, that can be taken without significantly increasing the effort beyond the established methods and with a more realistic accounting of the adsorbate entropy.

In the NH_3 Pt system, we couple four DOFs—v_{umb}, R_x, R_y, T_z—to hindered translation parallel to the Pt surface. The frequencies of these DOFs change most when moving along the hindered translation coordinate from the on-top binding site to the hopping transition state (see Table 2). Then, in analogy to the toy system described above, we write $Q_{\mathrm{ad}}^{\mathrm{cTST}}$ in the following form:

$$\begin{gathered} Q_{\mathrm{ad}}^{\mathrm{cTST}} = \left(\prod_{i=1}^{5} Q_{v_i}^{\mathrm{qHO}}\right) Q_{C_3}^{\mathrm{rot}} \frac{2\pi m k_{\mathrm{B}}T}{h^2} \times \\ \int_{-a/2}^{a/2} \int_{-b/2}^{b/2} Q_{v_{\mathrm{umb}}}^{\mathrm{qHO}}(x,y) Q_{R_x}^{\mathrm{qHO}}(x,y) Q_{R_y}^{\mathrm{qHO}}(x,y) Q_{T_z}^{\mathrm{qHO}}(x,y) \exp\left(-\frac{U_{n,\gamma}(x,y)}{k_{\mathrm{B}}T}\right) \mathrm{d}x\mathrm{d}y \left(\frac{Q_{T_x}^{\mathrm{qHO}}}{Q_{T_x}^{\mathrm{cHO}}}\right)\left(\frac{Q_{T_y}^{\mathrm{qHO}}}{Q_{T_y}^{\mathrm{cHO}}}\right). \end{gathered} \tag{S26}$$

The first six terms before the integral are identical to those used in uTST and represent vibrational partition functions that are independent of x and y. As a consequence of coupling, the minimum energy pathway emerging from a linear combination of multiple normal modes, cannot be described by hindered translational frequencies of the initial state, as we have done in uTST approach. The in-plane interaction potential, $U_{n,\gamma}(x,y)$ is parametrized similarly as described for uTST with the difference that here $f(\xi; n, \gamma)$ are fitted to the shape of the minimum energy path for hopping obtained from CI-NEB. See Figure S7, which shows the DFT minimum energy path as crosses and the best fit $f(\xi; n = 4.5, \gamma = 0.64)$ as a black solid line. The configuration integral now includes x- and y-dependent vibrational partition functions for the four DOFs whose frequencies change most dramatically when NH_3 translates from the on-top site to the diffusion transition state, according to DFT calculations.

These four partition functions are then parametrized as follows:

$$Q_{v_i}^{\mathrm{qHO}}(x,y) = \frac{1}{1 - \exp\left(-\frac{h v_i(x,y)}{k_B T}\right)}, \tag{S27}$$

with

$$v_i(x,y) = v_{i,\mathrm{bri}} + \left(v_{i,\mathrm{top}} - v_{i,\mathrm{bri}}\right) \exp\left(-\frac{x^2 + y^2}{\sigma_v^2}\right). \tag{S28}$$

$v_i(x,y)$ now describes the softening of the modes as the NH_3 molecule moves away from the atop binding site. Note, that we only use DFT frequency information for two structures: $v_{\mathrm{i,top}}$ for the top site and $v_{i,\mathrm{bri}}$ for the bridge site, which is the TS for hopping. The scaling with the in-plane coordinates is done assuming radial symmetry around the top site, which is a sensible approximation due to a localized bond formation between NH_3 and the top site of Pt. We have optimized σ_v such that $v(x,y)$ at the bridge site does not deviate by more than 0.1% from v_{bri} and yields good agreement with the temperature dependence of the desorption rate from Pt(111). The $v_i(x,y)$ functions are shown as blue solid lines in Fig. S7. The

uncertainties of NH_3* binding energy, that were derived from the cTST fit to the data, emerge from uncertainties associated with σ_ν. We have investigated the sensitivity of derived binding energies to σ_ν values that appeared reasonable in shape ($\nu_i(x,y)$ with FWHM between 0.5 and 2.0 Å) and estimated the uncertainty based on this.

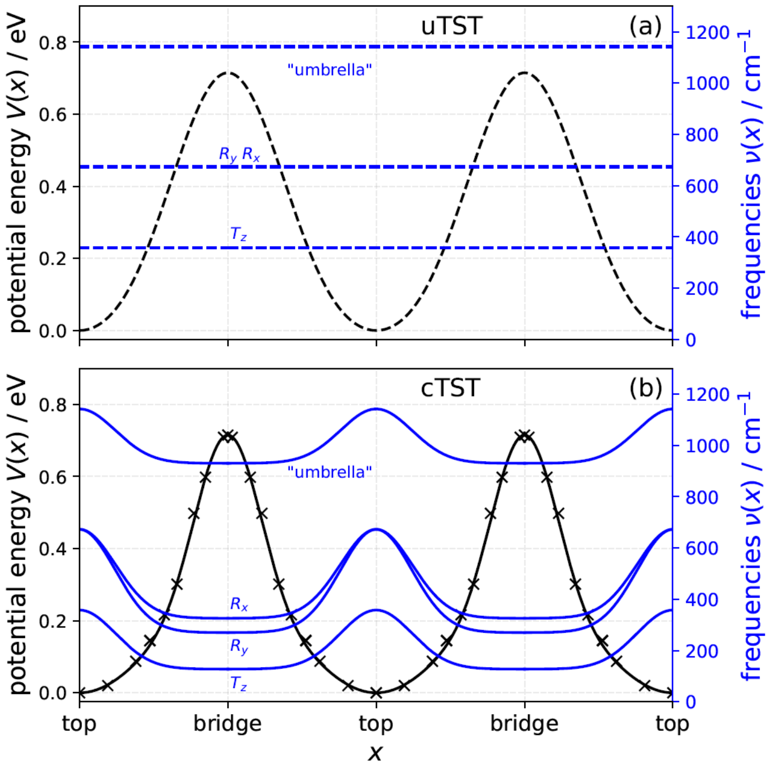

Figure S7: Coupling degrees of freedom in the TST of ammonia desorption and diffusion. (a) NH_3-Pt(111) interaction potential (black line) as a function of parallel translation x used to construct Q_{ad} within the uTST. It is parametrized based on the hindered translational frequency found from DFT at the top site and the experimentally derived hopping barrier. The blue dashed lines present the harmonic frequencies of vibrational modes that are associated with the molecule surface interaction. In this uncoupled picture the frequencies are independent of x. (b) NH_3-Pt(111) interaction potential (black line) as a function of parallel translation x used to construct Q_{ad} within the cTST. The crosses are the minimum energy path for diffusion derived from DFT calculations but scaled to match the experimental hopping barrier. Eq. S21 was used to generate the black solid line with optimized values of n and γ. The parallel translational dependence of the harmonic frequencies of four coupled modes is shown as blue solid lines. The region between two bridge sites characterizes the partition function for the adsorbate.

S6.3. Transition state for desorption

The desorbed gas-phase molecule is taken as the transition state for desorption in this entire work. Its partition function is given by:

$$Q^{\ddagger} = Q_{\mathrm{2D}}^{\mathrm{tr}} Q_{\mathrm{3D}}^{\mathrm{rot}} \left(\prod_{i=1}^{5} Q_{\nu_i}^{\mathrm{qHO}} \right) Q_{\mathrm{umb}}. \tag{S29}$$

Here, $Q_{\mathrm{2D}}^{\mathrm{tr}}$ is the partition function for free translation of a 2D ideal gas:

$$Q_{\mathrm{2D}}^{\mathrm{tr}} = \frac{2\pi m k_{\mathrm{B}} T}{h^2} A, \tag{S30}$$

with m being the mass of NH_3. A is the area of the Pt(111) unit cell, in which the partition function is defined. Note that translation normal to the surface is associated with the reaction coordinate in TST and is not part of $Q^{\ddagger}$.

$Q_{\mathrm{3D}}^{\mathrm{rot}}$ is the classical rotational partition function of a symmetric top:

$$Q_{\mathrm{3D}}^{\mathrm{rot}} = \frac{\sqrt{\pi}}{\sigma} \sqrt{\frac{(k_{\mathrm{B}} T)^3}{B^2 C}}, \tag{S31}$$

where B and C are the rotational constants and σ is the associated symmetry number.

The vibrational partition function can be accurately approximated by a simple quantum harmonic oscillator partition function $Q_{v_i}^{\mathrm{qHO}}$ for five DOFs: the symmetric and two asymmetric stretches as well as two asymmetric bends, conventionally referred to as $v_1 - v_5$. $Q_{v_i}^{\mathrm{qHO}}$ takes the usual form:

$$Q_{v_i}^{\mathrm{qHO}} = \frac{1}{1 - \exp\left(-\frac{h v_i}{k_\mathrm{B} T}\right)}. \tag{S32}$$

Here, v_i is the harmonic frequency of mode v_i. The umbrella mode is treated by explicit counting of the first ten vibrational levels, E_i^{umb}, to account for its complex quantum structure.

$$Q_{\mathrm{umb}} = \sum_{i=1}^{10} \exp\left(-\frac{E_i^{\mathrm{umb}}}{k_\mathrm{B} T}\right). \tag{S33}$$

All parameters needed to evaluate the partition function can be found in Refs. [13-15].

S6.4. Transition state for hopping

The transition state for hopping is described with the following partition function:

$$Q_{\mathrm{hop}}^{\ddagger} = \left(\prod_{i}^{5} Q_{v_i'}^{\mathrm{qHO}}\right) Q_{v_{umb}'}^{\mathrm{qHO}} Q_{R_x'}^{\mathrm{qHO}} Q_{R_y'}^{\mathrm{qHO}} Q_{T_z'}^{\mathrm{qHO}} Q_{C_3}^{\mathrm{rot}} \frac{\sqrt{2\pi m k_\mathrm{B} T}}{h} \int_{-b/2}^{b/2} \exp\left(-\frac{h(y)}{k_\mathrm{B} T}\right) \mathrm{d}y \left(\frac{Q_{y'}^{\mathrm{qHO}}}{Q_{y'}^{\mathrm{cHO}}}\right). \tag{S34}$$

The first nine partition functions describes the internal NH_3* vibrations, the frustrated rotations and translation (along the surface normal) as quantum harmonic oscillator with TS frequencies from Table 2. NH_3*'s rotation around its symmetry axis is assumed to be free. Since we find a rather low hindered translational frequency along the y-coordinate (x is taken as the reaction coordinate) we treat it by a hindered translator. Based on DFT generated harmonic frequencies, we estimate the shape of $h(y)$ using the commonly applied model potential energy function from Ref. [9]. We also include the low temperature correction to the partition function, previously described in Section 8.6.1.

We emphasize that this partition function does not take coupling of different DOFs into account. Including coupling would require vibrational frequencies of NH_3 along y while x would be fixed at the TS. Including coupling corrections would result in a somewhat higher value of the partition function than offered by the present approach.

S7. Extraction of First-Order rate constants from the desorption rates

We determine the first-order rate constants for NH_3 desorption from Pt(332) and the fast component in Pt(111) by fitting the flux $f(t)$ vs. residence time t using a function with two contributions,

$$f(t) = a \times \mathrm{DS}(t) + b \times \mathrm{TD}(t, k_{\mathrm{des}}), \tag{S35}$$

comprising direct scattering (DS) and a trapping-desorption (TD) with k_{des} as the desorption constant. The DS contribution has the temporal shape of the incident molecular beam, while the TD contribution is an exponential decay convoluted with the molecular beam temporal profile. For Pt(332) analysis, the full trace is fitted with Eq. S35, while for Pt(111) we cut the slow part off for the fit. The corresponding fits to the kinetic traces are shown in Fig. S8, while the fitted rate constants are shown in Fig. 3(a) (as red circles and black crosses) of the main text.

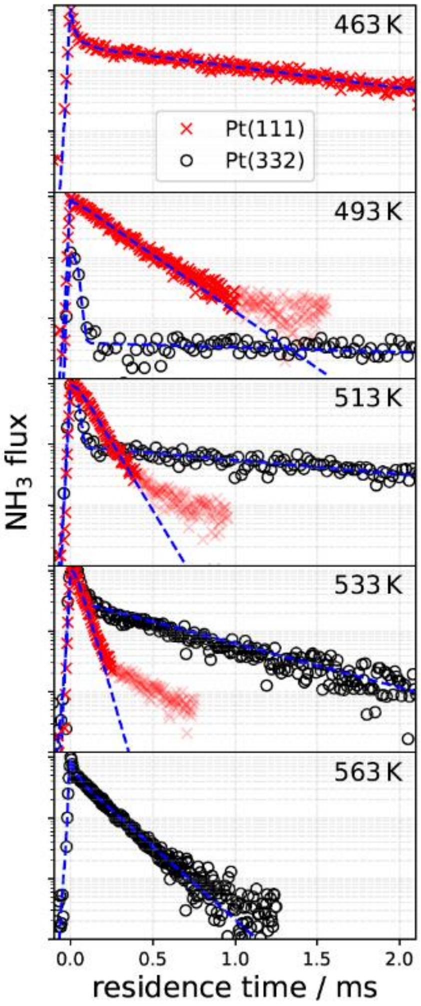

Figure S8: Desorption rate analysis with first-order rate constant extraction. Data from Pt(332), black circles, are fitted completely with Eq. S35, while Pt(111) data, red crosses, are fitted partially, with the excluded region shown in light red. The fits of Eq. S35 to the individual kinetic traces are shown by the blue dashed lines.

S8. Terrace-assisted desorption model from stepped surfaces

Here we derive the formula for the terrace-assisted desorption (TAD) model given in Eq. 4 of the main text. We assume a steady state between NH_3 at steps and terraces including the possibility to desorb from terraces and steps. An effective first-order desorption rate constant for NH_3 desorption from a stepped surface can be derived. We assume the following processes:

$$\mathrm{NH}_{3,\mathrm{T}}^{*} \underset{k_\mathrm{h}^\mathrm{S}(1-\mu)}{\overset{k_\mathrm{h}^\mathrm{T}\mu}{\rightleftarrows}} \mathrm{NH}_{3,\mathrm{S}}^{*} \,, \tag{S36}$$

$$\mathrm{NH}_{3,\mathrm{T}}^{*} \overset{k_\mathrm{d}^\mathrm{T}}{\rightarrow} \mathrm{NH}_3 \,, \tag{S37}$$

$$\mathrm{NH}_{3,\mathrm{S}}^{*} \overset{k_\mathrm{d}^\mathrm{S}}{\rightarrow} \mathrm{NH}_3 \,, \tag{S38}$$

where μ reflects the step density of the crystal. We define the total desorption rate and the effective first-order desorption rate constant as follows:

$$\frac{\mathrm{d}[\mathrm{NH}_3]}{\mathrm{d}t} = k_\mathrm{d}^\mathrm{T}\left[\mathrm{NH}_{3,\mathrm{T}}\right] + k_\mathrm{d}^\mathrm{S}[\mathrm{NH}_{3,\mathrm{S}}] \equiv k_\mathrm{eff}[\mathrm{NH}_{3,\mathrm{S}}] \tag{S39}$$

Since NH_3 has a big energy preference for steps, after a short induction period, the total desorption rate will be characteristic of the concentration pool at steps with the corresponding effective desorption rate constant. Assuming steady-state for $\left[\mathrm{NH}_{3,\mathrm{T}}\right]$, which is justified as shown in Section 3.5.2 of the main text we obtain:

$$\left[\mathrm{NH}_{3,\mathrm{T}}\right]_t = \frac{k_\mathrm{h}^\mathrm{S}(1-\mu)}{k_\mathrm{h}^\mathrm{T}\mu + k_\mathrm{d}^\mathrm{T}}\left[\mathrm{NH}_{3,\mathrm{S}}\right]_t \,. \tag{S40}$$

Combining Eq. S39 and S40 we obtain k_eff, shown as Eq. 4 of the main text and below:

$$k_\mathrm{eff} = k_\mathrm{d}^\mathrm{S} + k_\mathrm{h}^\mathrm{S}(1-\mu)\frac{k_\mathrm{d}^\mathrm{T}}{k_\mathrm{h}^\mathrm{T}\mu + k_\mathrm{d}^\mathrm{T}} \,. \tag{4}$$

We emphasize that the present model is kept simple and thus works only at stepped surfaces and adsorbates with strong energy preference. The results of the TAD model are shown in Fig. S9 as a black solid line in excellent agreement with experiment rate constants ($\chi^2 = 2.9$) obtained for NH_3 desorption from Pt (332) (black crosses). We also compare the results of an earlier model from Serri, Tully and Cardillo[3] (STC model) where no direct step desorption was considered (black dashed line). Neglecting direct step desorption degrades the fit ($\chi^2 = 16.3$) somewhat underestimating the experimental rate constants.

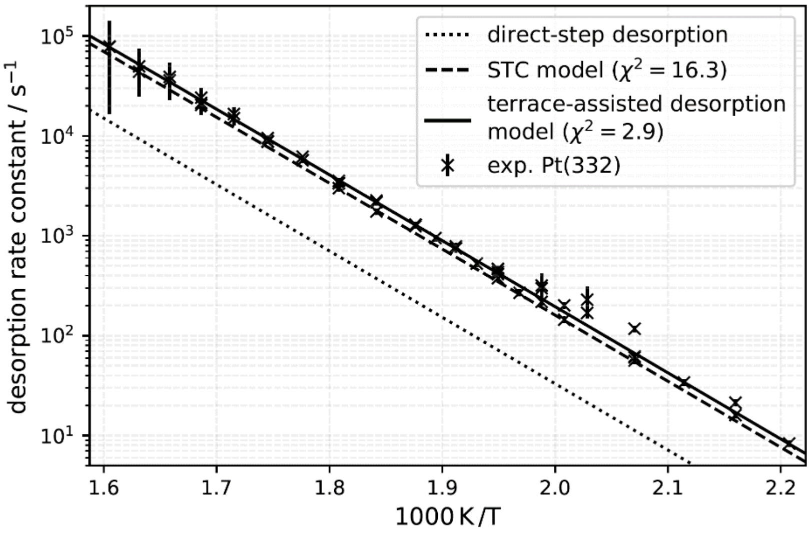

Figure S9: Comparison of experimental NH_3 desorption rate constants obtained with Pt (332) (black crosses) and three models for desorption from stepped surfaces. χ^2 in the legend is the reduced chi-square value.

S9. Coverage dependent desorption rates and adsorption isotherms

While the elementary reaction rate constants reported in this work were obtained for the low coverage limit, they can be used as a starting point to extend our understanding of the kinetics of NH_3 desorption from Pt. Here we explain how we have included the coverage dependence of the desorption rate constant from terraces and steps, based on the arguments elaborated in Section. 8.4.1 of the SI.

The coverage influences both the prefactor and the binding energy of the desorption rate constant:

$$k(T,\theta) = A(T,\theta)\exp\left(-\frac{E_0(\theta)}{k_B T}\right). \quad \text{(S41)}$$

From DFT calculations we find that NH_3 binding energy decreases linearly from $\theta = 0.06$ to 0.25 ML with a slope of $\alpha = -1.61$ eV/ML. We use the scaling of binding energy with coverage from DFT calculations, while using the experimentally derived zero-coverage binding energy ($E_{0,\mathrm{d}}^{\mathrm{T}} = 1.13 \pm 0.02$ eV). The resulting coverage dependent NH_3 binding energy at terraces is then the following

$$E_0(\theta)\ /\ \mathrm{eV} = 1.13 - 1.61 \times \left(\frac{\theta}{\mathrm{ML}}\right). \quad \text{(S42)}$$

We emphasize that the NH_3 saturation coverage of 0.25 ML is defined with respect to the Pt(111) facet.

We include the coverage dependence of the prefactor, $P_\theta(T)$, using the calculated harmonic frequencies of the adsorbate at 0.06 and 0.25 ML. We assume that the logarithm of the prefactor, which reflects the entropy difference between the initial state and the transition state, scales linearly with coverage and is described by:

$$\log_{10}\big(A(T,\theta)\big) = (1-4\theta)\log_{10}\big(P_{0.06}(T)\big) + 4\theta\log_{10}\big(P_{0.25}(T)\big). \quad \text{(S43)}$$

We note in passing that the prefactor calculated with frequencies at 0.06 ML is assumed to represent the prefactor in the zero-coverage limit. This introduces negligible changes in the results because the prefactor has only weak sensitivity to the assumptions of its scaling and the dominant contribution to the desorption rate comes from coverage dependence of NH_3's binding energy.

Using the coverage dependent desorption rate constant we simulate TPD spectra from previous work with the same experimental conditions, results of this simulation are shown in Fig. S10. We find overall good agreement with TPD spectra of the monolayer peak obtained from earlier studies.

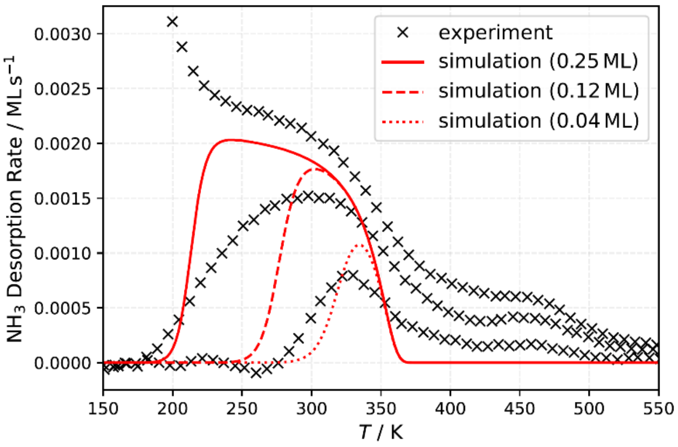

Figure S10: Previously measured NH_3 TPDs from Pt(111) from Ref. [16] (black crosses). Ref. [16] does not explicitly indicate the initial coverage in each of the three TPD spectra. The low temperature feature below 200 K is due to desorption of the multilayer only seen under conditions of the highest initial ammonia dose. At lower dosing, two TPD spectra are reported that represent desorption of the NH_3 monolayer at different initial coverages. The shoulder seen at 450 K emerges most likely from desorption influenced by steps. The red lines show TPD simulations of NH_3 desorption from Pt(111) based on Eq. S41 at three initial NH_3 coverages (see legend). It is clear that at shape of the TPD is strongly dependent on initial coverage—this reflects the influence of adsorbate-adsorbate interactions. The simulation captures this behavior reasonably well, validating our approach.

The adsorption isotherms are simulated using the diffusion-desorption kinetic model described in Section 8.4.2 of the SI. The stationary adsorption flux (at each spatial element i) is described by the Hertz-Knudsen equation:

$$F_{\mathrm{NH_3}} = \frac{p_{\mathrm{NH_3}}}{\sqrt{2\pi m_{\mathrm{NH_3}} k_{\mathrm{B}} T}}, \quad \text{(S44)}$$

instead of the molecular beam dosing function. For the chosen range of p_{NH_3} and T the rate equations are propagated in time until the stationary-state is established, from which the coverages at steps and terraces is derived. For simulation of the KB model[17] we use a surface with only terrace sites (as assumed by the KB model) in which the coverage independent NH_3 desorption rate constant is described by:

$$\frac{k_{\mathrm{d}}^{\mathrm{KB}}}{\mathrm{s}^{-1}} = 10^{9.74} \exp\left(-\frac{0.63\ \mathrm{eV}}{k_{\mathrm{B}}T}\right). \tag{S45}$$

The simulation results are shown in Figure 6 of the main text.

S10. Additional References

1. Borodin, D.; Rahinov, I.; Shirhatti, P. R.; Huang, M.; Kandratsenka, A.; Auerbach, D. J.; Zhong, T.; Guo, H.; Schwarzer, D.; Kitsopoulos, T. N.; Wodtke, A. M., Following the microscopic pathway to adsorption through chemisorption and physisorption wells. *Science* **2020,** *369* (6510), 1461-1465.

2. Guthrie, W. L.; Sokol, J. D.; Somorjai, G. A., The Decomposition of Ammonia on the Flat (111) and Stepped (557) Platinum Crystal-Surfaces. *Surf Sci* **1981,** *109* (2), 390-418.

3. Serri, J. A.; Tully, J. C.; Cardillo, M. J., The influence of steps on the desorption kinetics of NO from Pt(111). *The Journal of Chemical Physics* **1983,** *79* (3), 1530-1540.

4. Crank, J., *The Mathematics of Diffusion*. Clarendon Press: Oxford, 1975; Vol. 2.

5. King, D. A., Surface-Diffusion of Adsorbed Species - a Review. *J Vac Sci Technol* **1980,** *17* (1), 241-247.

6. Borodin, D.; Golibrzuch, K.; Schwarzer, M.; Fingerhut, J.; Skoulatakis, G.; Schwarzer, D.; Seelemann, T.; Kitsopoulos, T.; Wodtke, A. M., Measuring Transient Reaction Rates from Nonstationary Catalysts. *ACS Catal* **2020,** *10* (23), 14056-14066.

7. Novell-Leruth, G.; Valcarcel, A.; Clotet, A.; Ricart, J. M.; Perez-Ramirez, J., DFT characterization of adsorbed NHx species on Pt(100) and Pt(111) surfaces. *J Phys Chem B* **2005,** *109* (38), 18061-18069.

8. Garcia-Hernandez, M.; Lopez, N.; Moreira, I. D.; Paniagua, J. C.; Illas, F., Ab initio cluster model approach to the chemisorption of NH3 on Pt(111). *Surf Sci* **1999,** *430* (1-3), 18-28.

9. Sprowl, L. H.; Campbell, C. T.; Arnadottir, L., Hindered Translator and Hindered Rotor Models for Adsorbates: Partition Functions and Entropies. *J Phys Chem C* **2016,** *120* (18), 9719-9731.

10. Borodin, D.; Rahinov, I.; Fingerhut, J.; Schwarzer, M.; Hörandl, S.; Skoulatakis, G.; Schwarzer, D.; Kitsopoulos, T. N.; Wodtke, A. M., NO Binding Energies to and Diffusion Barrier on Pd Obtained with Velocity-Resolved Kinetics. *J Phys Chem C* **2021,** *125* (21), 11773-11781.

11. Jørgensen, M.; Grönbeck, H., Adsorbate Entropies with Complete Potential Energy Sampling in Microkinetic Modeling. *The Journal of Physical Chemistry C* **2017,** *121* (13), 7199-7207.

12. Bajpai, A.; Mehta, P.; Frey, K.; Lehmer, A. M.; Schneider, W. F., Benchmark First-Principles Calculations of Adsorbate Free Energies. *ACS Catal* **2018,** *8* (3), 1945-1954.

13. Nguyen, H. L.; Gulaczyk, I.; Kreglewski, M.; Kleiner, I., Large amplitude inversion tunneling motion in ammonia, methylamine, hydrazine, and secondary amines: From structure determination to coordination chemistry. *Coordin Chem Rev* **2021,** *436*.

14. Herzberg, G., *Electronic spectra and electronic structure of polyatomic molecules*. Van Nostrand: 1966; p 609.

15. Shimanouchi, T., *Tables of Molecular Vibrational Frequencies*. NSRDS: 1972; p 15.

16. Mieher, W. D.; Ho, W., Thermally Activated Oxidation of NH3 on Pt(111) - Intermediate Species and Reaction-Mechanisms. *Surf Sci* **1995,** *322* (1-3), 151-167.

17. Kraehnert, R.; Baerns, M., Kinetics of ammonia oxidation over Pt foil studied in a micro-structured quartz-reactor. *Chem Eng J* **2008,** *137* (2), 361-375.
